# Facile preparation of sulfonium peptide and protein probes for selective crosslinking of methyllysine readers†

**DOI:** 10.1039/d4sc05886k

**Published:** 2024-12-12

**Authors:** Kun Zou, Jinyu Yang, Yingxiao Gao, Feng Feng, Mingxuan Wu

**Affiliations:** a Department of Chemistry, Zhejiang University 310027 Hangzhou Zhejiang Province China wumingxuan@westlake.edu.cn; b Department of Chemistry, School of Science, Westlake University 310030 Hangzhou Zhejiang Province China; c Westlake Laboratory of Life Sciences and Biomedicine 310024 Hangzhou Zhejiang Province China; d Institute of Natural Sciences, Westlake Institute for Advanced Study 310024 Hangzhou Zhejiang Province China

## Abstract

Sulfonium is an electrophilic and biocompatible group that is widely applied in synthetic chemistry on small molecules. However, there have been few developments of peptide or protein-based sulfonium tools. We recently reported sulfonium-mediated tryptophan crosslinking and developed NleS^+^me2 (norleucine-dimethylsulfonium) peptides as dimethyllysine mimics that crosslink site-specific methyllysine readers. Therefore, sulfonium probes show great potential for investigating methyllysine readers and other aromatic cage-containing proteins. However, the current synthesis is not very efficient and is limited to peptide probes that, in many cases, cannot mimic protein–protein interactions. In addition to peptidyl conjugates that are valuable for reader identification, there are unavoidable methyl conjugates as side products. As a result, a robust method to prepare peptide and protein sulfonium tools with great crosslinking reactivity and selectivity is highly desirable. Here, we report a cysteine alkylation method to introduce site-specific sulfonium at protein level with excellent yield. In addition to dimethylsulfonium, we also developed cyclic sulfonium warheads that enhanced peptidyl conjugate selectivity. The method thus made it possible to prepare nucleosome probes in which LEDGF and NSD2, as H3K36 methylation readers were readily crosslinked. We thus believe this method will accelerate the development of sulfonium peptide and protein tool sets for broad applications in chemical biology studies.

## Abstract 2

A robust method is reported to introduce site-specific sulfonium warheads to peptides and proteins by cysteine alkylation. This method enables the preparation of nucleosome probes that crosslink to H3K36 methylation readers LEDGF and NSD2.

## Graphical abstract

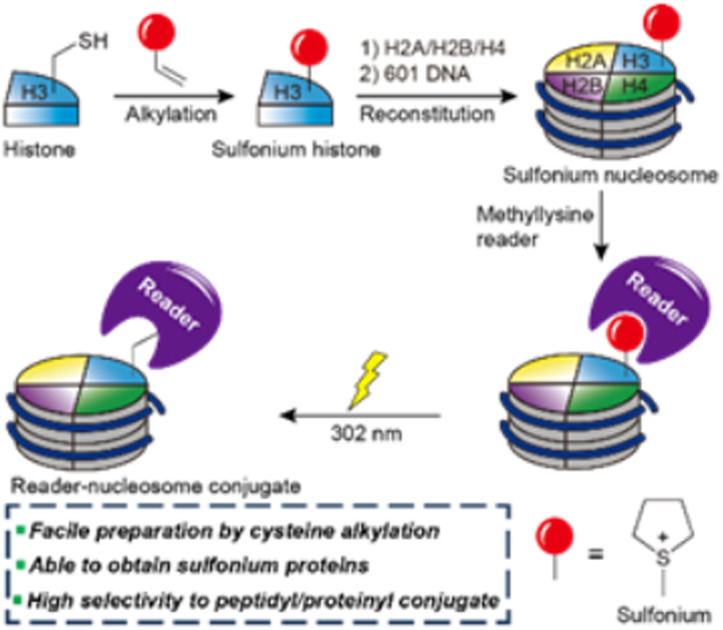

## Introduction

Lysine methylation is an important posttranslational modification. Histone lysine methylation regulates the chromatin structure and epigenetics.^1,2^ In addition, nonhistone lysine methylation controls diverse cellular processes such as MAP3K2 methylation^3,4^ and p53 methylation^5–7^ that drive cancer. Reader proteins, which bind to site-specifically modified protein motifs, are significant for downstream regulation upon lysine methylation.^8,9^ For example, CBX1 (a chromobox protein homolog, also known as heterochromatin protein HP1β) binds to histone H3 with K9 methylation and promotes the formation of heterochromatin and gene silencing.^10,11^ BPTF (bromodomain PHD finger transcription factor) contains a reader domain of H3K4 methylation that promotes gene transcription.^12,13^ Therefore, the identification and investigation of new methyllysine readers are essential to understanding the biological roles of lysine methylation at the molecular level.

**Fig. 1:**
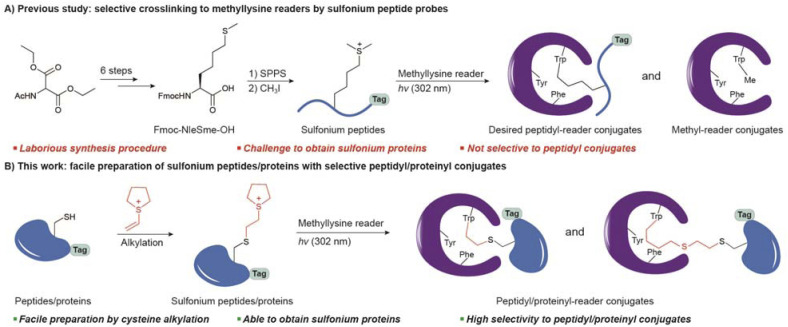
Overview of the previous strategy and the new strategy in this work to prepare sulfonium probes that selectively crosslink methyllysine readers. (A) In the previous method, sulfonium peptides were prepared from solid-phase peptide synthesis (SPPS) followed by *S*-methylation. The peptide probes mimic methyllysine and crosslink to tryptophan in a binding pocket. A peptidyl conjugate could be enriched by an affinity tag for downstream analysis, but the methyl conjugate was unavoidable. (B) In this study, sulfonium could be introduced by cysteine alkylation and the resulting peptides and proteins can also crosslink to the binding readers. In addition, cyclic sulfonium enables high selectivity to peptidyl/proteinyl conjugates.

Covalent probes are powerful for identifying proteins of interest (POI) because covalent linkage allows efficient enrichment of POI from complicated cellular samples.^14–16^ Probes that contain photoreactive groups are widely used,^17,18^ but laborious synthesis is required to optimize the placement of diazirine or BPA (*p*-benzoyl-l-phenylalanine) for efficient crosslinking. In addition, it is more synthetically challenging to prepare protein probes that target reader proteins.^19^ We recently reported a sulfonium-mediated crosslinking strategy so that NleS^+^me2 probes bind to readers as Kme2 peptide mimics and selectively crosslink to tryptophan inside the binding pocket (Fig. 1A).^20^ Because NleS^+^me2 plays roles in both binding and crosslinking, the design and synthesis are more straightforward.

However, there are some limitations to the current sulfonium method. First, sulfonium probes are limited to peptides with low synthesis yield. To introduce the unnatural amino acid residue, 6 steps are needed to prepare Fmoc-L-NleSme-OH as a building block for solid-phase peptide synthesis (SPPS). After peptide synthesis, extra *S*-methylation by iodomethane is necessary to yield NleS^+^me2 peptides.^21^ Second, sulfonium peptides may not mimic proteins in cellular environments. For example, histone H3K36 is proximate to DNA in the nucleosome structure,^22,23^ so an H3K36 methylation reader cannot bind a peptide well. Third, the crosslinking selectivity is not optimal. Peptidyl conjugates are desirable products for enrichment *via* affinity tags on peptides. However, irradiation also leads to a methyl-conjugate that reduces the efficiency of downstream applications (Fig. 1A).

Chemoselective modification of proteins is a major strategy for preparing site-specifically modified proteins.^24–26^ Due to its unique reactivity, cysteine is the most targeted residue for reactions, including it being a methyllysine mimic,^27^ a methylarginine mimic,^28^ and an acetyllysine mimic.^29–31^ Given the electrophilic nature of sulfonium, we envision that such a warhead may be incorporated into peptides and proteins by the nucleophilic thiol of cysteine. We found some clues in the reported methods of sulfonium synthesis and bioconjugation.^32–37^ Vinyl sulfonium has shown alkylation capability to thiol in previous studies.^32^ A very recent study reported vinyl aryl sulfonium for selective cysteine alkylation on proteins, but the sulfonium intermediate is designed to leave from proteins. The resulting episulfonium on the protein is functionalized by additional nucleophiles.^38^ Here we report a cysteine alkylation method to introduce sulfonium as a dimethyllysine and NleS^+^me2 mimic at specific sites (Fig. 1B). Such a method makes sulfonium peptides and proteins much more synthetically accessible and could be widely used to develop sulfonium probes for tryptophan site-specific crosslinking and the study of methyllysine readers.

## Results and discussion

### Development of a robust alkylation method to prepare Nle_C_S^+^me2 peptides

**Fig. 2:**
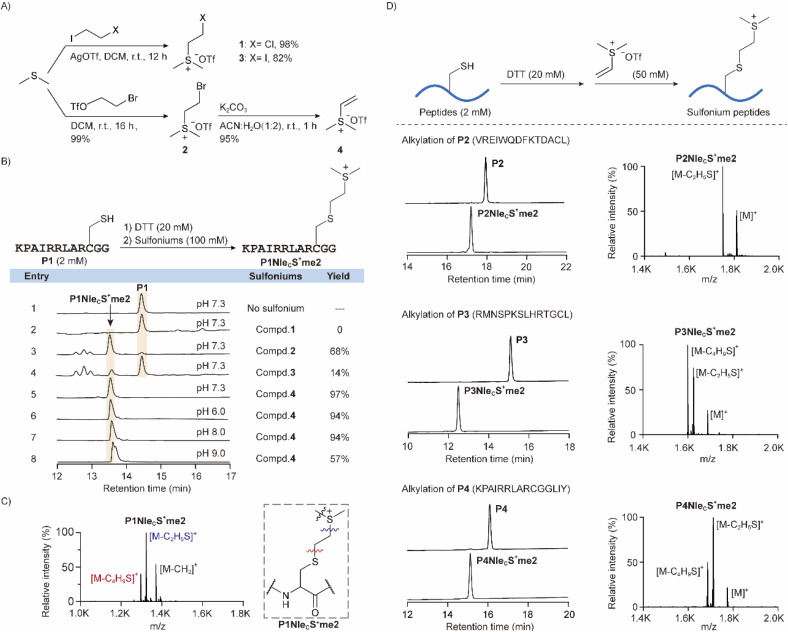
Establishment of cysteine alkylation method to prepare sulfonium peptides. (A) Preparation of sulfonium compounds 1–4 as alkylating agents. (B) Screening of P1 alkylation conditions using sulfonium compounds 1–4. (C) Characterization of P1 alkylation product by high resolution mass spectroscopy (HR-MS). Fragments were indicated from different C–S bond cleavages in mass spectrometry. (D) More peptides were alkylated by 4 with excellent yield. The reaction mixtures were analysed by HPLC and HR-MS.

As we aim to introduce sulfonium to cysteine on free peptides and proteins, the alkylating agent should meet a few requirements. First, the agent should be electrophilic enough for alkylation by nucleophilic thiol on cysteine. Second, the reactivity should be highly selective to cysteine as peptides and proteins contain diverse functional groups. Third, the reaction should proceed under physiological conditions so that the protein products will still be well-folded. Fourth, the alkylation products should be structurally close to NleS^+^me2 for selective crosslinking to tryptophan inside a methyllysine-binding pocket in the readers. As a result, we designed several sulfonium compounds 1–4 for initial screening (Fig. 2A). Sulfonium compounds 1 and 3 were prepared from dimethylsulfide and haloethyl iodide reagents. Alternatively, bromide compound 2 was prepared with bromoethyltriflate. A subsequent elimination reaction of 2 under basic conditions yielded vinyl sulfonium 4.^32,39^

With the agents in hand, we started to characterize reactivity and selectivity using a cysteine-containing peptide P1 (Fig. 2B). P1 was pre-treated with DTT as a general procedure for cysteine alkylation to break the possible disulfide bonds of cysteine-containing samples formed during storage (Fig. S5†)^27,40^ and was subsequently treated with sulfonium alkylation agents. By HPLC analysis, compound 2 yielded a significant amount of desired product, as confirmed by mass spectrometry (Fig. 2C), but there were several additional peaks from side products. Compounds 1 and 3, with different halogens, however, were much less active. Compound 4 exhibited the best performance with excellent yield. Therefore, we further characterized the reactivity of sulfonium 4 at different pH values (Fig. 2B, entries 5–8), times (Fig. S3A†), and temperatures (Fig. S3B†). Another product peak was observed at pH 9 and was later indicated to be a dialkylated product by mass spectrum analysis (Fig. S3C†). We suggest that the extra alkylation occurs on the *N*-amine or lysine side chain due to the enhanced nucleophilic substitution reactivity of amine at pH 9. Therefore, we explored potential amine reactivity using Boc-Lys-OH with free ε-amine. A clear alkylation product from sulfonium 4 was identified at pH 9, but no such reaction happened at pH 7.3 (Fig. S4†). The reaction rate was generally fast as 100% conversion could be achieved within 15 min at 27 °C. We thus later selected another three peptides that contain all functional groups of natural amino acids, and all the alkylation reactions of compound 4 were robust and clean (Fig. 2D).

Michael addition is a widely used method to selectively modify cysteine on peptides and proteins, but there is an instability issue due to the retro-Michael reaction.^41,42^ To characterize the stability of the sulfonium from cysteine alkylation, we prepared a glutathione conjugate 6 (Fig. S12†) to measure stability using ^1^H NMR and HPLC. It exhibited excellent stability after 48 h in both pH 7.3 buffer and pH 9.5 buffer (Fig. S21–23†).

**Fig. 3:**
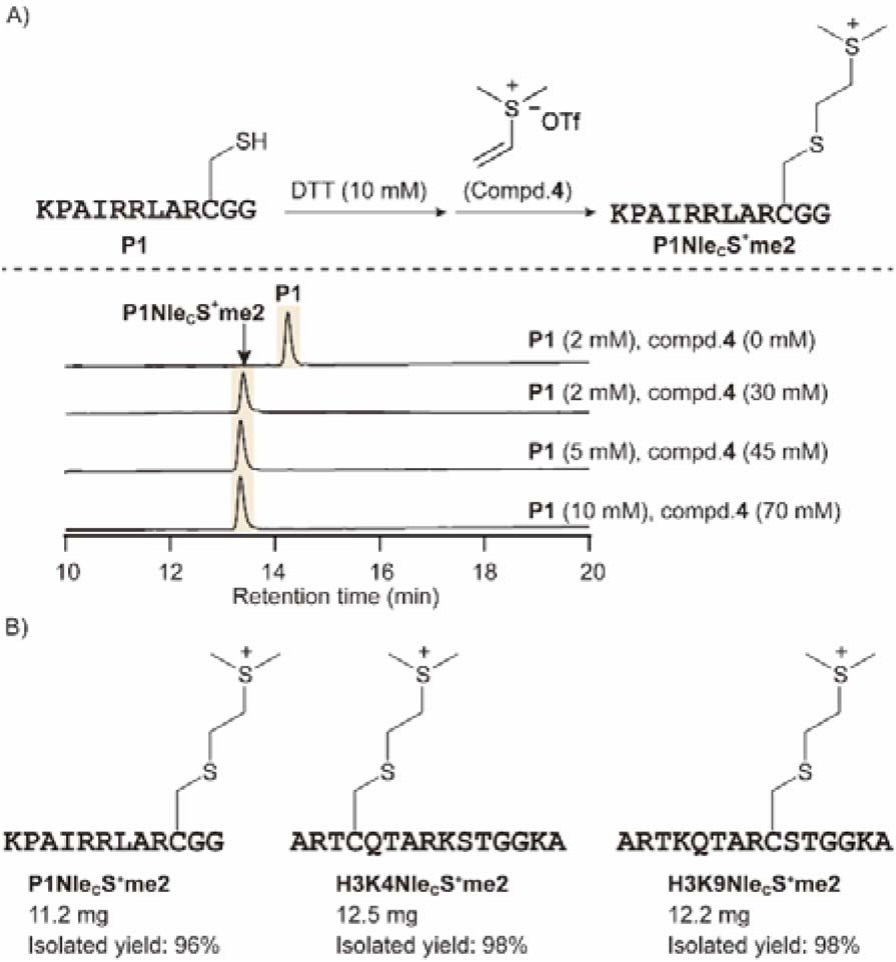
Optimization of conditions for scaling-up the preparation of Nle_C_S^+^me2 peptides. (A) P1 was readily converted to P1Nle_C_S^+^me2 with an increase in peptide concentration, as illustrated by HPLC. (B) Several peptides were alkylated at over 10 mg scale with excellent isolated yields.

As the analytical yield with the vinyl sulfonium was promising, we next optimized the peptide concentration and alkylating agent 4 (Fig. 3A). 10 mM peptide was also readily converted to sulfonium product, and thus we can scale up the synthesis in a more concentrated mixture. We next applied peptide P1 at 10 mg scale and obtained the product P1Nle_C_S^+^me2 with 96% isolated yield. Similarly, H3K4Nle_C_S^+^me2 and H3K9Nle_C_S^+^me2 peptides were obtained with excellent isolated yield, that will be studied for methyllysine reader crosslinking (Fig. 3B).

### Nle_C_S^+^me2 is a good mimic of NleS^+^me2 for tryptophan crosslinking

**Fig. 4:**
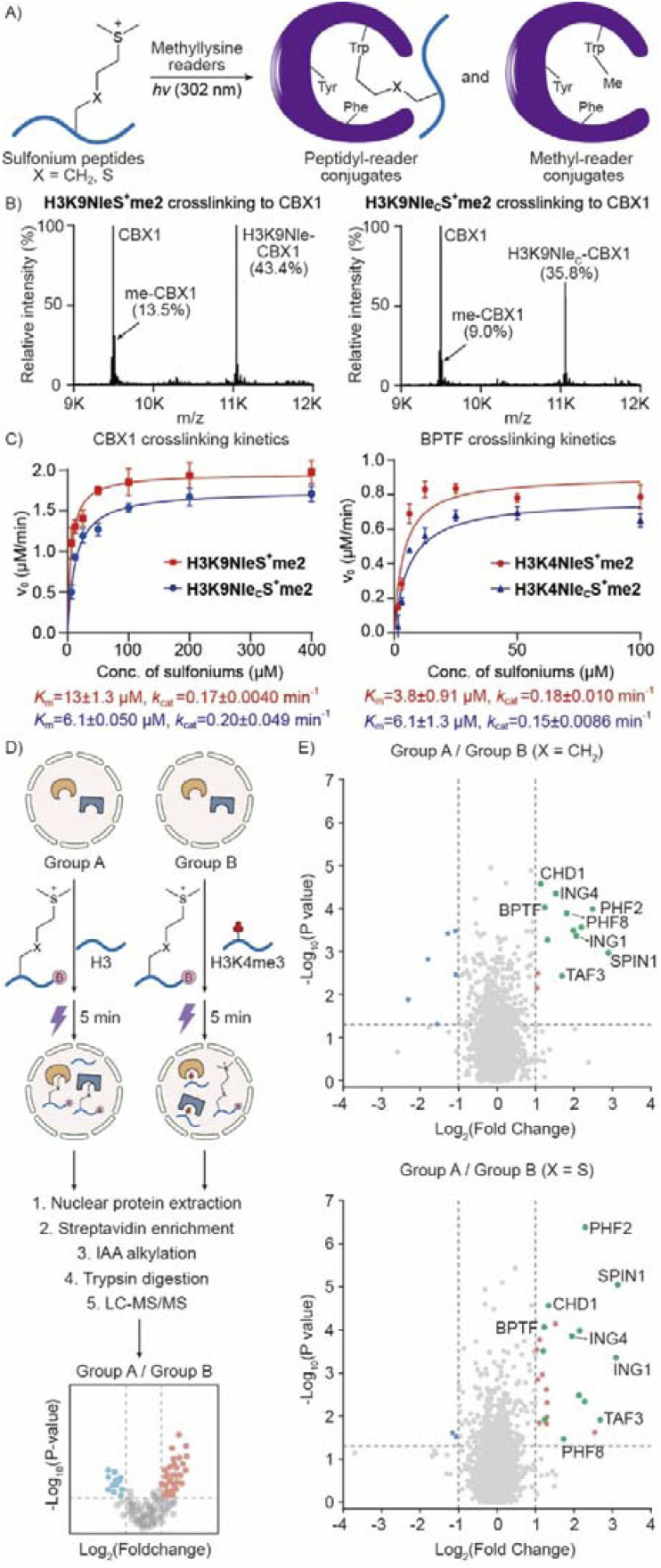
Comparison of reader crosslinking activities between NleS^+^me2 and Nle_C_S^+^me2. (A) Sulfonium peptides selectively crosslink tryptophan in a methyllysine reader binding pocket under UV-B irradiation. (B) HR-MS of photo-crosslinking mixtures of 100 μM sulfonium peptides and 10 μM CBX1 for 15 min. The analytical yields were quantified from integration of the mass peaks. (C) 10 μM CBX1 or 5 μM BPTF was treated with sulfonium probes at various concentrations for 3 min of photo-crosslinking. The products were quantified by mass spectrometry and the calculated initial reaction rates *v*_0_ were used for a plot of sulfonium peptide concentrations by the Michaelis–Menten equation; error bars represent mean ± SE (*n* = 3 crosslinking replicates). (D) Extracted HeLa cell nuclei were treated with 50 μM sulfonium peptide with a desthiobiotin/biotin tag and 500 μM binding competition peptides (unmodified H3 peptide in group A and H3K4me3 peptide in group B) for crosslinking under UV irradiation. Crosslinked proteins were enriched by streptavidin and processed for quantitative mass spectrometry. (E) Volcano plots of the crosslinked proteins from H3K4NleS^+^me2 or H3K4Nle_C_S^+^me2 probes with different levels of competition by unmodified or Kme3 peptides. The hits for known H3K4 methylation readers in the plot with a fold change of ≥2 and *P* value of ≤ 0.05 are shown as green dots. Shared reader hits in both volcano plots are highlighted by the name.

With the establishment of a method for sulfonium peptide preparation, we next wondered whether Nle_C_S^+^me2 is a good mimic of NleS^+^me2. We thus set up photo-crosslinking reactions using several methyllysine readers (Fig. 4A). Generally, the reaction yields of peptidyl conjugate and methyl conjugate between Nle_C_S^+^me2 and NleS^+^me2 peptides were very close (Fig. 4B and S15†). To compare the two sulfonium compounds in more detail, we carried out a kinetic analysis of crosslinking to CBX1 and BPTF. The correlation between reaction rate and sulfonium peptide concentration was quite similar (Fig. 4C). Regression analysis with the Michaelis–Menten equation indicated very close *K*_m_ and *k*_cat_ values. Furthermore, we applied H3K4NleS^+^me2 and H3K4Nle_C_S^+^me2 to extracted cell nuclei and volcano plots demonstrated that similar groups of protein hits were selectively crosslinked and enriched by the two sulfonium peptides with biotin tags (Fig. 4D and E). Thus, we concluded that Nle_C_S^+^me2 is a good mimic of NleS^+^me2 for reader crosslinking from individual recombinant proteins to the proteome.

### Cyclic sulfonium improved peptidyl-conjugation

From our previous studies, we found that photo-crosslinking between sulfonium peptide probes and readers led to two conjugates (Fig. 4A).^20^ Once sulfonium accepts an excited single electron from indole, we suggest that homolysis of the C–S bond yields a sulfide and an alkyl radical to crosslink Trp*^+^. Therefore, peptidyl and methyl conjugates were both obtained (Fig. 4B and S15†). Because only the peptidyl conjugate could be enriched by an affinity tag on the peptide, methyl conjugation reduces the efficiency for following protein ID.

**Fig. 5:**
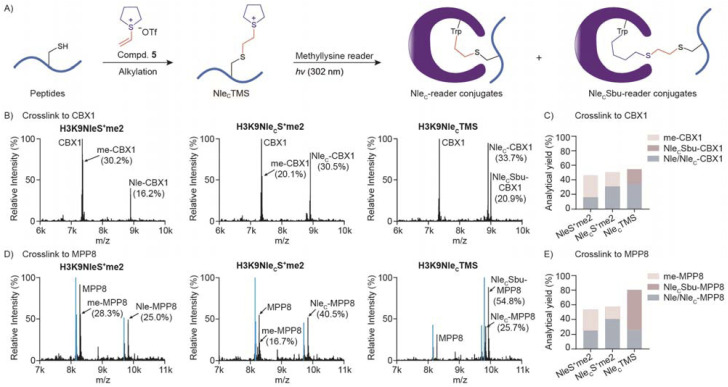
Cyclic sulfonium peptides improved peptidyl conjugation yields. (A) Cyclic Nle_C_TMS sulfonium peptides were prepared by cysteine alkylation and treated with readers for photo-crosslinking that resulted in two types of peptidyl conjugate. (B–E) HR-MS of the reaction mixtures of 3 μM CBX1 in (B) or MPP8 in (D) and 100 μM H3K9NleS^+^me2, H3K9Nle_C_S^+^me2 or H3K9Nle_C_TMS under UV-B irradiation for 5 min (CBX1) or 20 min (MPP8). The recombinant MPP8 also contains a proteoform with loss of *N*-methionine that is labelled as cyan peaks in (D). The analytical yields were quantified by mass peak integration and are summarized in bar graphs, as shown in (C) and (E).

We thus designed cyclic sulfonium to overcome this limitation because any possible C–S homolytic cleavage would only result in a peptidyl conjugate. In addition, there were cyclic amine structures in methyllysine reader inhibitors, which indicate tolerance of such a group in the reader binding pocket.^43,44^ We thus made efforts to prepare a series of vinyl cyclic sulfonium compounds. Probably due to high strain, three- or four-member ring products could not be obtained. Instead, vinyl cyclic sulfonium with five- or six-member rings were readily synthesized and applied to cysteine alkylation to yield Nle_C_TMS (tetramethylene sulfide) and Nle_C_PMS (pentamethylene sulfide) (Fig. 5A, S8 and 9†). We also prepared a glutathione conjugate 7 that demonstrated the same stability as Nle_C_S^+^me2 in buffers (Fig. S13 and S24–26†).

We next compared dimethylsulfonium and cyclic sulfonium for crosslinking reactivity and selectivity. H3K9Nle_C_TMS resulted in two expected crosslinking products, Nle_C_-CBX1 conjugate and Nle_C_Sbu-CBX1 conjugate, with a combined analytical yield of peptidyl conjugate that is significantly higher than the yield using H3K9NleS^+^me2 and H3K9Nle_C_S^+^me2 (Fig. 5B and C). In the case of MPP8 (M-phase phosphoprotein 8), the peptidyl conjugate yield with H3K9Nle_C_TMS exhibited over a 3-fold increase compared to H3K9NleS^+^me2 (Fig. 5D and E). We next compared Nle_C_TMS and Nle_C_PMS crosslinking activities to CBX1 and MPP8. Both cyclic sulfonium compounds exhibited a close overall analytical yield of peptidyl conjugates, and the Nle_C_-reader was a preferred product over a Nle_C_Sbu-reader or Nle_C_Spe-reader (Fig. S16†). Further kinetic analysis revealed faster Nle_C_-reader conjugation but slower Nle_C_Sbu conjugation by Nle_C_TMS (Fig. S17†). Overall, cyclic sulfonium-mediated crosslinking avoids a methyl conjugate with much superior peptidyl conjugation to dimethylsulfonium for downstream protein ID applications.

### Development of sulfonium nucleosome probes for reader crosslinking

Peptides are frequently used to study posttranslational modifications that mimic the partial structure of proteins such as histone tails.^45–47^ However, for histone sites in a globular domain that have interactions with other core histones and DNA, peptide-based studies are not ideal.^40,48^ As the vinyl sulfonium exhibited high reactivity and selectivity on peptides, we think the reaction would also be robust on proteins to prepare protein sulfonium probes.

**Fig. 6:**
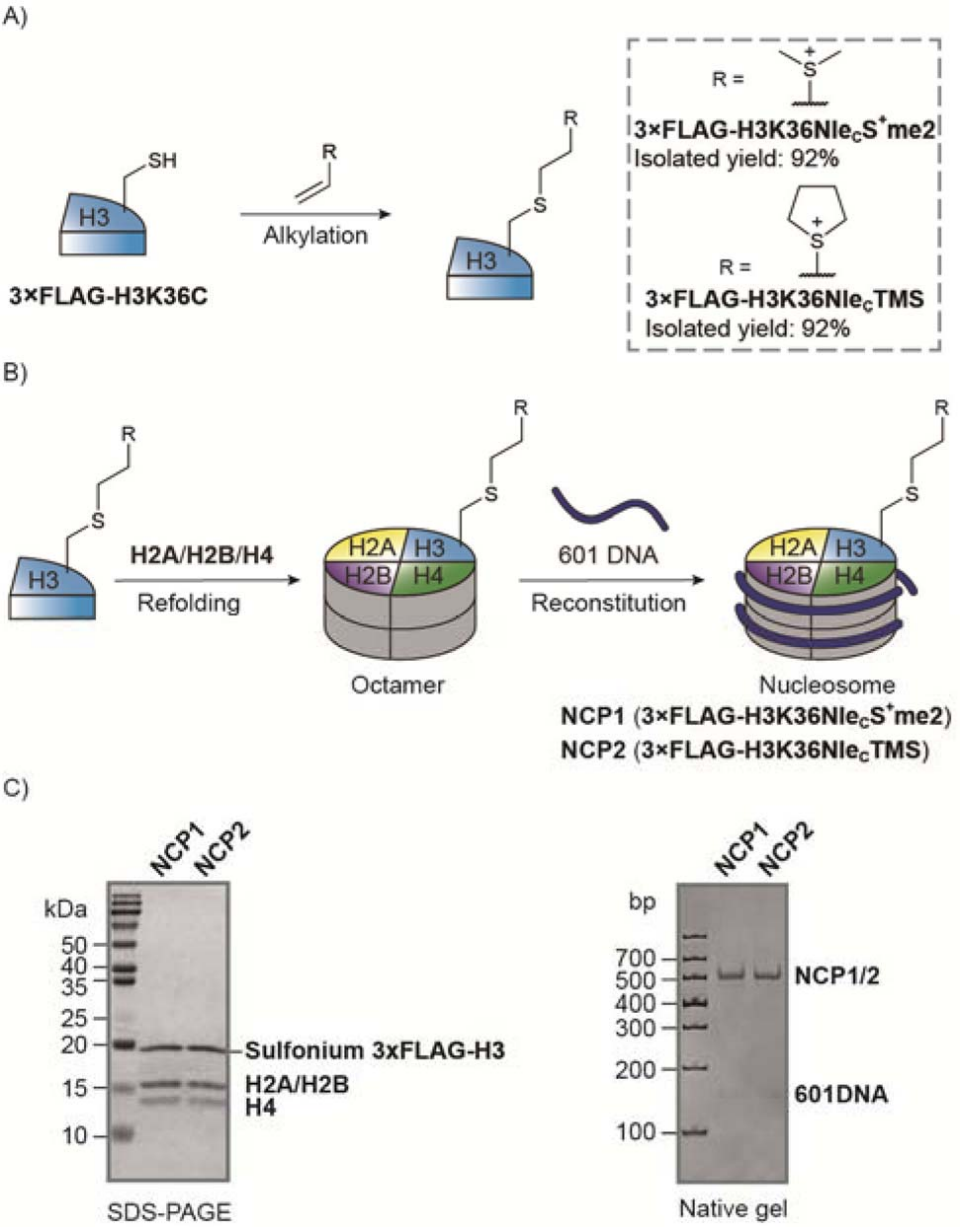
Preparation of sulfonium nucleosomes. (A) Histone 3×FLAG-H3K36Nle_C_S^+^me2 and 3×FLAG-H3K36Nle_C_TMS were prepared by alkylation of recombinant H3K36C with excellent isolated yields. (B) Scheme toward sulfonium nucleosomes (NCP1 and NCP2). (C) Characterization of NCP1 and NCP2 by SDS-PAGE and native gel.

We prepared a recombinant *X. laevis* histone 3xFLAG-H3K36C mutant for vinyl sulfonium alkylation. Both sulfonium 4 and 5 yielded the desired products with 92% isolated yield at multiple mg scale (Fig. 6A). We next used the sulfonium-containing histone H3 to prepare nucleosome core particles (NCP) NCP1 and NCP2 (Fig. 6B). The yield of octamer refolding and nucleosome reconstitution were the same as for histone 3xFLAG-H3K_c_36me3. In addition, the mobile shifts of the nucleosomes were the same as for H3K_c_36me3 nucleosome NCP3 on a native PAGE gel (Fig. 6C and S20†). Thus, the sulfonium group is well tolerated through the nucleosome preparation. To confirm the stability of sulfonium, we monitored NCP2 using mass spectrometry and the sulfonium group was intact over 45 days in storage buffer at 4 °C (Fig. S27†).

**Fig. 7:**
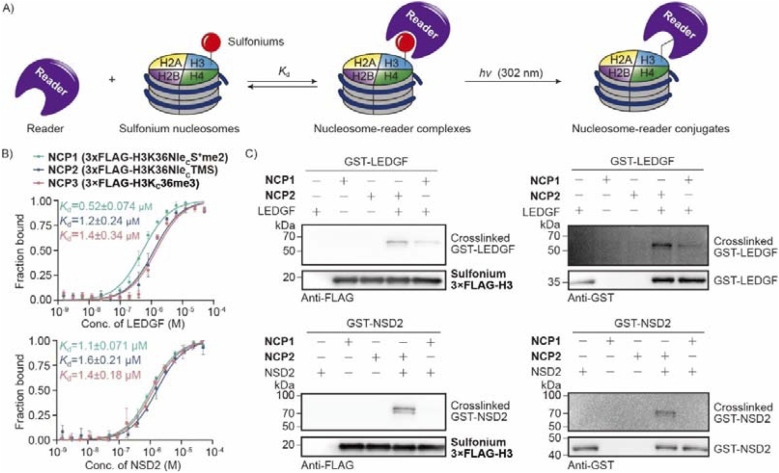
Sulfonium nucleosomes crosslink to H3K36me3 readers. (A) Scheme of sulfonium nucleosomes binding and crosslinking to H3K36me3 readers. (B) Binding affinities of Kme3 or sulfonium nucleosomes to readers were measured by microscale thermophoresis (MST); error bars represent mean ± SE (*n* = 3 replicates). (C) H3K36me3 readers LEDGF and NSD2 were treated with Nle_C_S^+^me2 or Nle_C_TMS nucleosomes under irradiation. The crosslinking products were visualized with western blot using anti-FLAG or anti-GST antibodies due to the FLAG tag on nucleosomes and the GST tag on readers.

H3K36 methylation is an important PTM and there are several reported readers that interact with H3K36me3 by aromatic cages.^49,50^ In addition, the readers interact closely with DNA that is proximate to H3K36. As histone H3 peptides cannot mimic the local chemical environment in chromatin, we aimed to prepare nucleosome probes with sulfonium at H3K36 for reader crosslinking. We expressed recombinant PWWP domains of LEDGF and NSD2 that are well studied as H3K36 methylation readers and later measured binding affinities to the nucleosomes. The data demonstrated that sulfonium NCP1 and NCP2 are good mimics of H3K_c_36me3 NCP3 with close *K*_d_ values (Fig. 7A and B). Next, the mixtures of NCP and readers were irradiated using UV-B and analyzed using western blot (Fig. 7A and C). The data demonstrated crosslinking activity between sulfonium NCP and the readers. Moreover, NCP2 which contains cyclic sulfonium, exhibited much better peptidyl conjugation than NCP1 (Fig. 7C). This result further confirms the improved efficiency of reader crosslinking by employment of cyclic sulfonium. Overall, the sulfonium-containing proteins are readily available by cysteine alkylation and nucleosome probes are active for crosslinking the site-specific readers.

## Conclusions

Here we have developed a facile method to prepare sulfonium-containing peptides and proteins by cysteine alkylation. Vinyl sulfoniums are simple to prepare and exhibit robust reactivity and selectivity to cysteine. Therefore, a milligram scale of peptides and proteins with diverse sulfonium is easily achievable. Moreover, the resulting Nle_C_S^+^me2 and derivatives are good mimics of methyllysine for site-specific tryptophan crosslinking inside the binding pocket of readers. In addition to sulfonium peptide probes, we demonstrated that the method enables sulfonium-containing nucleosome probes that mimic chromatin to crosslink readers that interact with globular domains. In addition, cyclic sulfonium enhanced crosslinking to readers for higher efficiency for protein ID. Consequently, we believe that sulfonium probes formed with vinylsulfonium could be widely used for tryptophan crosslinking and aromatic cage investigations.

## Data availability

The data that support the findings of the study are available in the ESI† of this article.

## Author contributions

M. W. conceived the idea and secured funding acquisition. K. Z. established the alkylation method, prepared sulfonium peptides and proteins, and performed kinetic experiments. J. Y. characterized crosslinking using recombinant readers and cell nuclei and prepared nucleosomes and readers with investigation of the binding and crosslinking. Y. G. prepared some reader proteins and F. F. synthesized Fmoc-NleSme-OH. K. Z. and J. Y. drew the figures and wrote the manuscript with the guidance of M. W., and all authors contributed to the editing of the manuscript.

## Conflicts of interest

There are no conflicts to declare.

## Supplementary Material

SC-OLF-D4SC05886K-s001
