# Supplementary material for: Facile preparation of sulfonium peptide and protein probes for selective crosslinking of methyllysine readers
Source: Chem Sci. 2024 Dec 12;16(4):1849–56. doi: 10.1039/d4sc05886k (PMC11665613; doi:10.1039/d4sc05886k)
Supplement: SC-016-D4SC05886K-s001 [file SC-016-D4SC05886K-s001.pdf]

Supplementary Information (SI) for Chemical Science.
This journal is © The Royal Society of Chemistry 2024

Supporting Information

Facile preparation of sulfonium peptide and protein probes for selective crosslinking of methyllysine readers

Kun Zou,[#a,b] Jinyu Yang, [#a,b] Yingxiao Gao,[b] Feng Feng,[c] Mingxuan Wu*[b,c,d]

[a]Department of Chemistry, Zhejiang University, Hangzhou 310027, Zhejiang Province, China

[b]Department of Chemistry, School of Science, Westlake University, Hangzhou 310030, Zhejiang Province, China

[c]Westlake Laboratory of Life Sciences and Biomedicine, Hangzhou 310024, Zhejiang Province, China

[d]Institute of Natural Sciences, Westlake Institute for Advanced Study, Hangzhou 310024, Zhejiang Province, China

#These authors contributed equally to this work

*Correspondence to: wumingxuan@westlake.edu.cn

Content

1 General information 2
1.1 Materials and reagents 2
1.2 HPLC, mass spectrometry and NMR 2
2 Chemical synthesis 4
3 Peptide synthesis 7
3.1 General procedure for peptide synthesis 7
3.2 Sequence and MS data of peptides 7
4 Establishment of cysteine alkylation method 9
4.1 Screen of various sulfoniums 9
4.2 Screen of equivalents of compound 4 10
4.3 Screen of reaction conditions 11
4.4 Analysis of amine alkylation at pH 9.0 12
4.5 Significance of DTT in pretreatment 13
4.6 Peptide expansion 14
5 Preparation of sulfonium peptides in large scale 16
5.1 Optimization of sulfonium concentrations 16
5.2 Scale up synthesis of sulfonium peptides 16
6 Preparation of sulfonium peptides 17
7 General procedure of crosslinking between sulfonium probes and readers...24
7.1 Crosslink CBX1, MPP8, BPTF with sulfonium peptides 25
7.2 Crosslink CBX1, MPP8 with H3K9NleS$^+$me2, H3K9Nle$_C$S$^+$me2, H3K9Nle$_C$TMS 25
7.3 Crosslink CBX1, MPP8 with H3K9Nle$_C$TMS, H3K9Nle$_C$PMS 26
8 Crosslinking kinetics comparison between sulfonium probes and readers....27
8.1 Kinetics study of the crosslinking between sulfonium probes and CBX1 27
8.2 Kinetics study of the crosslinking between sulfonium probes and BPTF 28
9 Proteome-wide crosslinking experiment 29
10 Preparation of sulfonium proteins 31
11 Preparation of sulfonium nucleosomes 33
12 Stability study of sulfonium peptides and nucleosomes 35
12.1 Stability study of sulfonium peptides 35
12.2 Stability study of sulfonium nucleosomes 39
13 Expression and purification of LEDGF and NSD2 40
14 Binding affinity between readers and sulfonium nucleosomes 41
15 Crosslinking to H3K36me3 readers with sulfonium nucleosomes 42
16 HR-MS and NMR data 43
16.1 MS data of sulfoniums (ESI-TOF) 43
16.2 MS data of peptides (MALDI-TOF and ESI-TOF) 43
16.3 NMR of sulfoniums (^{1}H NMR and ^{13}C NMR) 44
17 References 49

1 General information

1.1 Materials and reagents

Reagents for chemical synthesis were purchased from Adamas, Sigma-Aldrich, Energy chemistry, and J&K. Solvents were purchased from Shanghai Titan Scientific Co., Ltd, including dichloromethane (DCM), methanol (MeOH), ethyl acetate (EA), petroleum ether (PE), diethyl ether, acetone, *N*,*N*-dimethyl-formamide (DMF), *N*-methyl-2-pyrrolidone (NMP), *N*,*N*-diisopropyl-carbodiimide (DIC) and ethyl cyanoglyoxylate-2-oxime (Oxyma). Trifluoroacetic acid (TFA, HPLC grade), triisopropylsilane (TIPS) and formic acid (FA) (LC-MS grade), acetonitrile (ACN, HPLC grade) were purchased from J&K. All Fmoc-AA-OH and Rink Amide-AM Resin (loading: 0.657 mmol/g) were purchased from GL Biochem (Shanghai), Ltd. Dithiothreitol (DTT), 2-mercaptoethanol (BME), guanidine hydrochloride (GdnHCl), glutathione (GSH), Boc-Lys-OH, and phosphate were purchased from VWR Life Science, J&K Scientific, Sangon Biotech, and Shanghai Titan Scientific Co., Ltd, respectively.

1.2 HPLC, mass spectrometry and NMR

The chemical compounds in this article were purified by SepaBean^{TM} machine T with irregular silica, and then characterized by NMR spectra with 500 MHz Solution NMR Spectrometer (Cryo Probe, Bruker, AVANCE NEO) and high-resolution mass spectra (HR-MS) (Waters/Synapt XS HDMS).

The alkylation reactions were analyzed and purified by reverse phase high performance liquid chromatography (RP-HPLC) (Waters, 1525 binary pump and 2489 UV/visible detector). The analysis of peptide alkylation was carried out with C18 column (XBridge Peptide BEH C18 column, 130 Å, 5 μm, 4.6 mm × 150 mm) while the protein alkylation was carried out with C4 column (XBridge Protein BEH C4 column, 300 Å, 3.5 μm, 4.6 mm × 150 mm). The flow rate of the mobile phase was at 1.0 mL/min with UV absorption at 220 nm and 254 nm. For the preparation of sulfonium peptides, they were purified with C18 column (XBridge Peptide BEH C18 column, 130 Å, 10 μm, 19 mm × 150 mm) at a flow rate of 10 mL/min, while the

sulfonium proteins were purified with C4 column (XBridge Protein BEH C4 column, 300 Å, 5 µm, 10 mm × 250 mm) at a flow rate of 5 mL/min with UV absorption at 220 nm and 238 nm. A binary mixture of solvent A (0.1% TFA in H_2O) and solvent B (0.1% TFA in acetonitrile) were used as mobile phase for the analysis and preparation of sulfonium peptides and proteins.

Peptides and proteins were characterized by high resolution mass spectra (HR-MS) (Waters/Synapt XS HDMS) and matrix-assisted laser desorption/ionization-time of flight (MALDI-TOF) (Bruker, Rapiflex) matrixed with α-cyano-4-hydroxycinnamic acid (HCCA).

2 Chemical synthesis

Synthesis of 2-chloro-ethyl-dimethyl-sulfonium triflate (**1**)

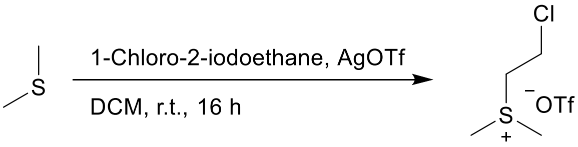

1

To a solution of dimethyl sulfide (147 μL, 2.0 mmol) in DCM (anhydrous) (8 mL) was added 1-chloro-2-iodoethane (219 mg, 2.4 mmol) and AgTfO (617 mg, 2.4 mmol), stirred at room temperature for 16 h. After finish of this reaction, sediment formed and was removed by filtration, and then the solvent was removed under vacuum condition. The crude was redissolved in 3 ml of acetone, followed by addition of 40 ml of cool diethyl ether to precipitate the dimethyl sulfonium. The target product precipitated after centrifuge at 4500 rpm for 5 min (4 °C), which was repeated twice. The crude was dried in vacuo to afford the yellow oil compound **1** (566 mg, 98%). ^{1}H NMR (500 MHz, acetone-d_6) δ 4.25 (dd, J = 6.7, 5.9 Hz, 2H), 4.07 (dd, J = 6.6, 5.9 Hz, 2H), 3.27 (s, 6H). ^{13}C NMR (126 MHz, acetone-d_6) δ 55.3, 34.4, 24.3. HR-MS (ESI-TOF): $[M]^+$ *m/z* found: 125.0206, calculated: 125.0186; $[M+Na]^+$ *m/z* found: 147.9356, calculated: 148.0186; $[M-CH_2]^+$ *m/z* found: 109.9061, calculated: 109.9952.

Synthesis of 2-bromo-ethyl-dimethyl-sulfonium triflate (**2**)

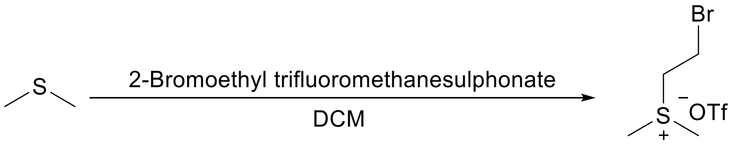

2

To a solution of dimethyl sulfide (220 μL, 3.0 mmol) in DCM (anhydrous) (5 mL) was added 2-bromoethyl trifluoromethanesulphonate (135 μL, 1.0 mmol), stirred at room temperature for 16 h. After finish of this reaction, solvent was removed under vacuum condition. The crude was redissolved in 3 ml acetone, followed by addition of 40 ml cool diethyl ether to precipitate the sulfonium product. The target product precipitated after centrifuge at 4500 rpm for 5 min (4 °C), which was repeated twice.

The crude was dried in vacuo to afford the brown oil compound **2** (302 mg, 99%). ^{1}H NMR (500 MHz, acetone-d_6) δ 4.28 – 4.10 (m, 2H), 4.10 – 4.01 (m, 2H), 3.26 (s, 6H). ^{13}C NMR (126 MHz, acetone-d_6) δ 45.4, 24.5, 23.9. HR-MS (ESI-TOF): $[M]^+$ *m/z* found: 168.9684, calculated: 168.9681; $[M-C_2H_5]^+$ *m/z* found: 140.9398, calculated: 140.9190; $[M-C_2H_5S]^+$ *m/z* found: 109.8502, calculated: 108.9476.

Synthesis of 2-iodo-ethyl-dimethyl-sulfonium triflate (**3**)

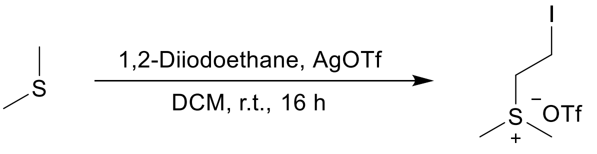

3

The method to prepare compound **3** was the same to that of compound **1** and brown oil product **3** was obtained (600 mg, 82%). ^{1}H NMR (500 MHz, acetone-d_6) δ 4.19 – 3.97 (m, 2H), 3.89 – 3.65 (m, 2H), 3.22 (s, 6H). ^{13}C NMR (126 MHz, acetone-d_6) δ 45.5, 24.1, -8.2. HR-MS (ESI-TOF): $[M]^+$ *m/z* found: 216.9545, calculated: 216.9542; $[M-C_2H_5]^+$ *m/z* found:188.9283, calculated:188.9031; $[M-C_2H_5S]^+$*m/z* found: 154.9387, calculated: 154.9358.

Synthesis of dimethylvinylsulfonium triflate (**4**)

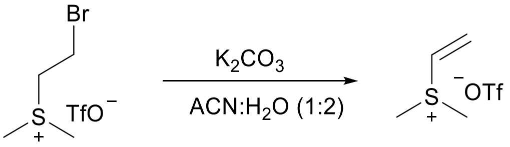

4

To a solution of compound **2** (1.0 mmol) in the mixed solvent (2 mL, ACN: H_2O 1:1) was added K_2CO_3(0.5 mmol in 1 mL H_2O), stirred at room temperature for 4 h. After finish of this reaction, solvent was removed under vacuum condition. The crude was redissolved in 3 mL acetone and the white salt was removed by filtration. The solvent was removed in vacuo to afford the yellow oil product **4** (275 mg, 95%). ^{1}H NMR (500 MHz, acetone-d_6) δ 7.01 (dd, J = 16.4, 9.2 Hz, 1H), 6.60 (dd, J = 16.3, 2.1 Hz, 1H), 6.52 (dd, J = 9.2, 2.1 Hz, 1H), 3.28 (s, 6H). ^{13}C NMR (126 MHz, acetone-d_6)

δ 134.3, 125.2, 26.8. HR-MS (ESI-TOF): $[M]^+$ *m/z* found: 89.0426, calculated: 89.0419; $[M-C_2H_5]^+$ *m/z* found: 62.0203, calculated: 61.9908.

***Synthesis of 1-vinyltetrahydro-1H-thiophenium triflate* (5)**

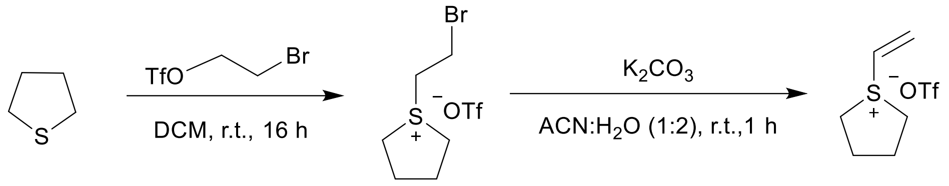

5

The method to prepare compound **5** was the same to that of compound **4** and brown oil product **5** was obtained (420 mg, 63.4%). ^{1}H NMR (500 MHz, acetone-d_6) δ 6.94 (dd, J = 16.3, 9.2 Hz, 1H), 6.56 (dd, J = 16.3, 2.0 Hz, 1H), 6.45 (dd, J = 9.2, 2.0 Hz, 1H), 4.01 – 3.91 (m, 2H), 3.81 – 3.70 (m, 2H), 2.58 – 2.48 (m, 2H), 2.48 – 2.37 (m, 2H). ^{13}C NMR (126 MHz, acetone-d_6) δ 133.9, 125.2, 46.9, 28.6. HR-MS (ESI-TOF): $[M]^+$ *m/z* found: 115.0587, calculated: 115.0576; $[M-C_2H_2]^+$ *m/z* found: 89.0426, calculated: 89.0341.

***Synthesis of 1-vinyltetrahydro-1H-thiopyranium triflate* (6)**

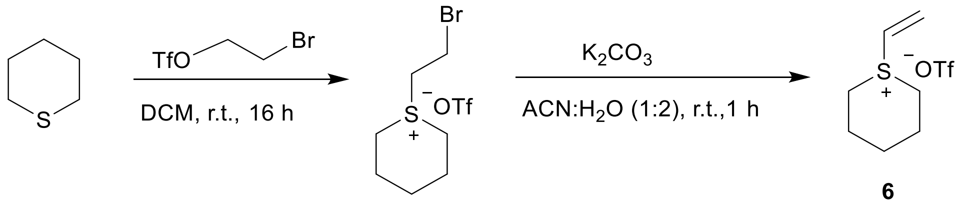

6

The method to prepare compound **6** was the same to that of compound **4** and brown oil product **6** was obtained (360 mg, 54.3%). ^{1}H NMR (500 MHz, acetone-d_6) δ 7.06 (dd, J = 16.5, 9.6 Hz, 1H), 6.67 – 6.49 (m, 3H), 3.84 (ddd, J = 12.5, 8.5, 3.4 Hz, 2H), 3.71 (ddd, J = 12.7, 8.5, 3.4 Hz, 2H), 2.23-2.33(m, 2H), 2.06 – 1.95 (m, 2H), 1.91 – 1.74 (m, 2H). ^{13}C NMR (126 MHz, acetone-d_6) δ 135.3, 121.8, 38.1, 22.1, 20.5. HR-MS (ESI-TOF): $[M]^+$ *m/z* found: 129.0749, calculated: 129.0732.

3 Peptide synthesis

3.1 General procedure for peptide synthesis

The synthesis of peptides in this article was performed based on standard fluorenyl methoxycarbonyl (Fmoc)-solid-phase peptide synthesis (SPPS) on a 0.1 mmol scale by a Liberty Blue 2.0 automated microwave peptide synthesizer (CEM Corp., U.S.A.).

The Rink Amide-AM resin initially was bubbled at room temperature with N_2 in DMF for 10 min to swell the resin. The synthesis of peptides was following the standard deprotection and coupling cycles. For the deprotection cycle, Fmoc group was removed with 4 mL of 4-methylpiperidine (20% in DMF) at 30 °C for 6 min, which was repeated twice. For the coupling step, 5 equivalents of Fmoc-AA-OH, 5 equivalents of DIC and 10 equivalents of Oxyma were added to the reaction vessel to couple the amino acids to resin at 80 °C for 4 min. 4 mL of DMF was used to wash the resin for three times after every Fmoc deprotection and amino acid coupling cycle. Amino acids were coupled to the resin in sequence by repeating the deprotection and coupling cycles. After completion of the peptide synthesis, the resin was washed with DMF, DCM and MeOH in sequence and air-dried under room temperature for further cleavage.

Peptides were cleaved from resin with cocktail (4 mL, TFA: TIPS: phenol: water 88:2:5:5) by rotating at room temperature for 3 h. The resin was removed and cold diethyl ether (36 mL) was added to the filtrate to precipitate the peptides, followed by centrifuge at 4500 rpm/min for 5 min. The sediment was washed with cold diethyl ether (40 mL) again and air-dried under room temperature to obtain the crude.

3.2 Sequence and MS data of peptides

1) **P1**, sequence: KPAIRRLARC GG-NH_2, HR-MS (MALDI-TOF): $[M+H]^+$ found: 1296.81, calculated: 1296.59.

2) **H3K4C**, sequence: ARTCQTARKS TGGKA-NH_2, HR-MS (MALDI-TOF): $[M+H]^+$ *m/z* found: 1534.82, calculated: 1534.74.

3) **H3K9C**, sequence: ARTKQTARCS TGGKA-NH_2, HR-MS (MALDI-TOF): $[M+H]^+$ *m/z* found: 1534.95, calculated: 1534.74.

4) **H3K4NleSme**, sequence: ARTNleSmeQTARKS TGGKA-NH_2, HR-MS (MALDI-TOF): $[M+H]^+$ *m/z* found: 1590.83, calculated: 1590.68.

5) **H3K9NleSme**, sequence: ARTKQTARNleSmeS TGGKA-NH_2, HR-MS (MALDI-TOF): $[M+H]^+$ *m/z* found: 1590.84, calculated: 1590.68.

6) **P2**, sequence: VREIWQDFKTDACL-NH_2, HR-MS (ESI-TOF): $[M+H]^+$ *m/z* found: 1722.96, calculated: 1722.85.

7)**P3**, sequence: RMNSPKSLHRTGCL-NH_2, HR-MS (ESI-TOF): $[M+H]^+$ *m/z* found: 1598.90, calculated: 1598.82.

8) **P4**, sequence: KPAIRRLARCGGLIY-NH_2, HR-MS (ESI-TOF): $[M+H]^+$ *m/z* found: 1686.08, calculated: 1686.01.

9) **H3K4CK16bio**, sequence: ARTCQTARKS TGGKA Kbio-NH_2, HR-MS (ESI-TOF): $[M+2H]^{2+}$ *m/z* found: 945.00, calculated: 945.61; $[M+3H]^{3+}$ *m/z* found: 630.68, calculated: 630.74.

4 Establishment of cysteine alkylation method

4.1 Screen of various sulfoniums

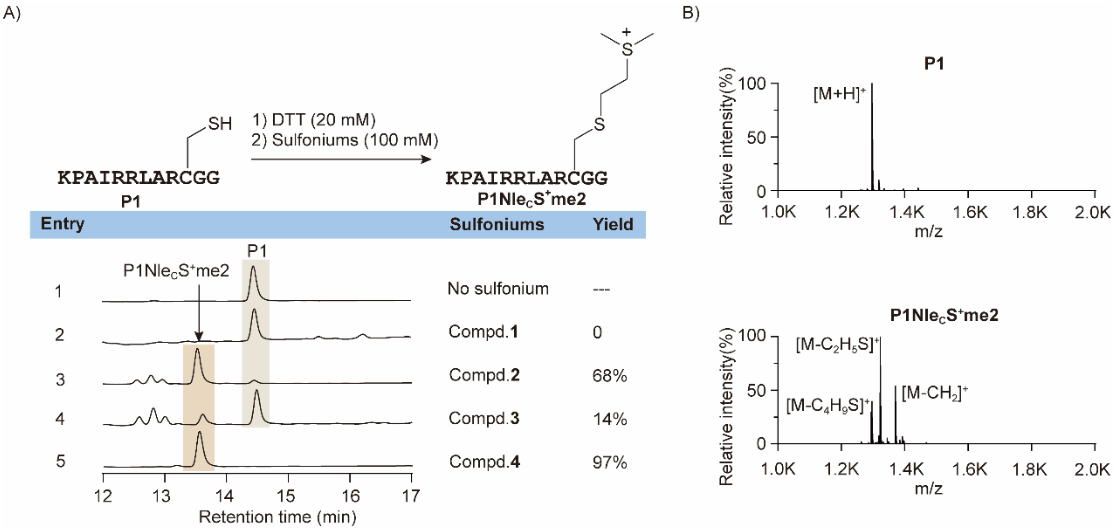

Figure S1 Screen of sulfoniums for the cysteine alkylation. (A) The alkylation reactions were analyzed by HPLC, **P1** and **P1Nle$_C$S$^+$me2** were marked by different colors. (B) **P1Nle$_C$S$^+$me2** was determined with MALDI-TOF: $[M-CH_2]^+$ *m/z* found: 1370.80, calculated: 1370.64; $[M-C_2H_5S]^+$ *m/z* found: 1324.80, calculated: 1324.82; $[M-C_4H_9S]^+$ *m/z* found: 1297.78, calculated: 1297.82.

Parallel experiments containing 5 groups were performed to determine the optimal sulfonium reagent.

At room temperature, 10.0 μL of **P1** (20 mM, 1.0 equiv.) in H_2O was added to 5 Eppendorf tubes (1.5 mL) and diluted with 76.0 μL of NaPi buffer (4 M GdnHCl, 0.1 M NaPi, pH 7.3) respectively. Subsequently, 4.0 μL of a DTT stock solution (0.5 M, 10 equiv.) in H_2O was added to the mixtures. These mixtures were vortexed for 3 seconds, transferred into a heating block pre-heated at 37 °C, and incubated for 1 h. Next, these mixtures were cooled by ice bath for 10 min, and then 10 μL of different sulfonium stock solutions (Compd.**1**-**4**) (1.0 M, 50 equiv.) in NMP were added at room temperature respectively. The reaction mixtures were vortexed for 3 seconds, transferred into a heating block pre-heated at 37°C, and incubated for 2 h. After finish of these reactions, 2.0 μL of a HCl stock solution (6.0 M) was added to quench these reactions. Finally, samples were prepared by centrifuge at 15000 rpm for 5 min and analyzed by LC with a gradient of 5%-40% B in 20 min, the retention time of

P1Nle$_C$S$^+$me2 is at 13.4 min. The purified fractions were collected and determined by ESI-TOF.

4.2 Screen of equivalents of compound 4

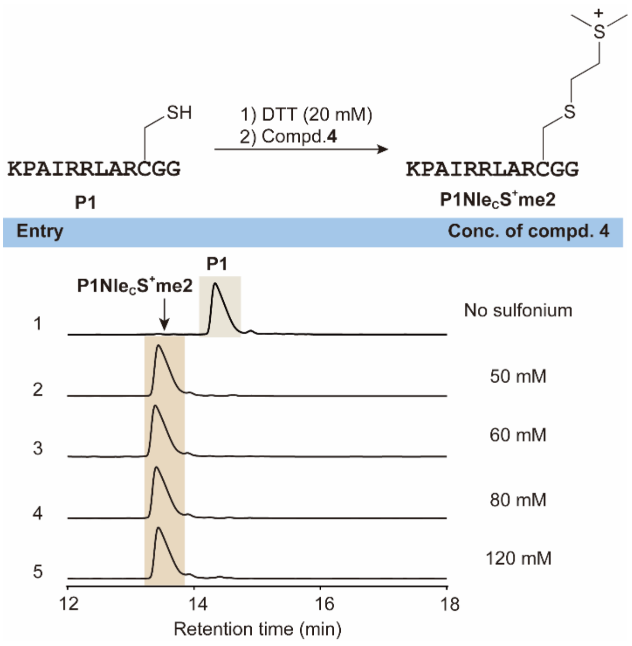

Figure S2 HPLC analysis of the alkylation reactions with different concentrations of compd.**4**. **P1** and **P1Nle$_C$S$^+$me2** were marked by different colors and labels.

At room temperature, **P1** (20 mM, 1.0 equiv.) in H_2O was added to 5 Eppendorf tubes (1.5 mL) and diluted with NaPi buffer (4 M GdnHCl, 0.1 M NaPi, pH 7.3) respectively. Subsequently, DTT stock solution (0.5 M, 10 equiv.) in H_2O was added to the mixtures. These mixtures were vortexed for 3 seconds, transferred into a heating block pre-heated at 37 °C, and incubated for 1 h. Next, these mixtures were cooled by ice bath for 10 min, and then compd.**4** stock solution (1.0 M, 0/25/30/40/60 equiv.) in NMP was added to these tubes at room temperature respectively. These reaction mixtures were vortexed for 3 seconds, transferred into a heating block pre-heated at 37°C, and incubated for 2 h. After finish of these reactions, 2.0 μL of a HCl stock solution (6.0 M) was added to quench these reactions. Finally, these samples were prepared by centrifuge at 15000 rpm for 5 min and analyzed by LC with a gradient of 5%-40% B in 20 min, the retention time of **P1Nle$_C$S$^+$me2** is at 13.4 min. The purified fractions were collected and determined by ESI-TOF.

4.3 Screen of reaction conditions

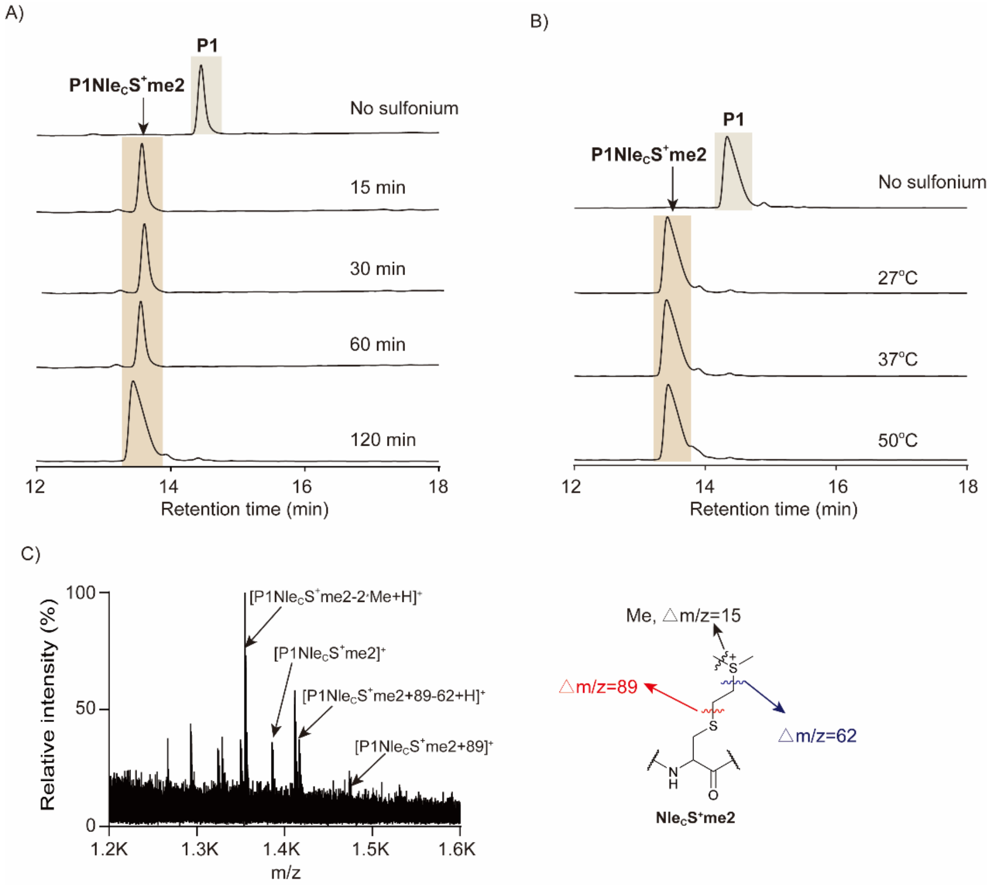

Figure S3 Influence of reaction time, temperature, and pH on the cysteine alkylation with compd.**4**. (A) The alkylation reactions were analyzed by HPLC at different time points. (B) The alkylation reactions were analyzed by HPLC at different temperatures. (C) HR-MS of the collected cysteine alkylation sample at pH 9.0, the ionic fragments were analyzed and marked by different colors. **P1** and **P1Nle$_C$S$^+$me2** were marked by different colors.

At room temperature, **P1** (20 mM, 1.0 equiv.) in H_2O was added to 3 Eppendorf tubes (1.5 mL) and diluted with NaPi buffer (4 M GdnHCl, 0.1 M NaPi, pH 7.3) respectively. Subsequently, DTT stock solution (0.5 M, 10 equiv.) in H_2O was added to the mixtures. These mixtures were vortexed for 3 seconds, transferred into a heating block pre-heated at 37 °C, and incubated for 1 h. Next, these mixtures were cooled by ice bath for 10 min, and then compd.**4** stock solution (1.0 M, 25 equiv.) in NMP was added to these tubes at room temperature respectively. These reaction mixtures were vortexed for 3 seconds, transferred into heating block pre-heated at 27/37/50°C, and

incubated at 27/37/50°C for 2 h. The following procedure for these reactions were the same to previous method.

To investigate the influence of reaction pH on this reaction, parallel experiments containing 3 groups were performed at pH 6.0/8.0/9.0, the reaction temperature is at 37°C. The following procedure for these reactions were the same to previous method.

To investigate the influence of reaction pH on this reaction, experiment was monitored at different time points. At room temperature, **P1** (20 mM, 1.0 equiv.) in H_2O was added to Eppendorf tube (1.5 mL) and diluted with NaPi buffer (4 M GdnHCl, 0.1 M NaPi, pH 7.3). Subsequently, DTT stock solution (0.5 M, 10 equiv.) in H_2O was added to the mixture. The mixture was vortexed for 3 seconds, transferred into a heating block pre-heated at 37 °C, and incubated for 1 h. Next, the mixture was cooled by ice bath for 10 min, and then compd.**4** stock solution (1.0 M, 25 equiv.) in NMP was added to the tube at room temperature. The reaction mixture was vortexed for 3 seconds, transferred into heating block pre-heated at 37°C, incubated at 37°C and monitored with HPLC after 15/30/60/120 min. The following procedure for this reaction was the same to the previous method.

4.4 Analysis of amine alkylation at pH 9.0

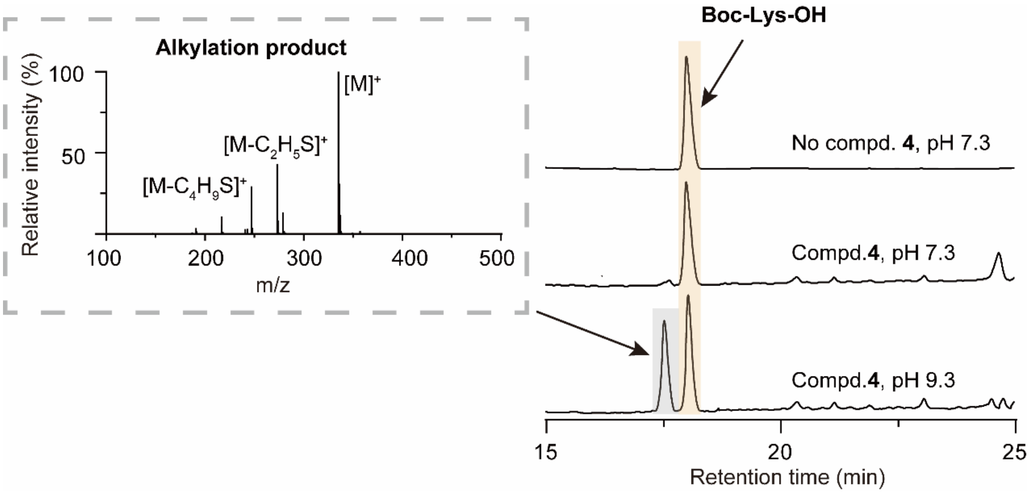

Figure S4 Analysis of the alkylation reactions at different pH levels. **Boc-Lys-OH** was alkylated with compound **4** at pH 7.3 and 9.3 and the reaction mixture was analyzed by HPLC and ESI-TOF. The alkylation product was determined by ESI-TOF and the ionic fractions were marked by labels.

Alkylation product: $[M]^+$ *m/z* found: 335.2017, calculated: 335.1999; $[M\text{-}C_2H_5S]^+$ *m/z* found: 273.1812, calculated: 273.1817; $[M\text{-}C_4H_9S]^{3+}$ *m/z* found: 254.9673, calculated: 245.1501.

At room temperature, 95 µL NaPi buffers (0.1 M NaPi, pH7.3), 91 µL NaPi buffers (0.1 M NaPi, pH7.3) and 91 µL carbonate buffers (0.1 M carbonate, pH9.3) were added to 3 Eppendorf tubes (A/B/C, 1.5 ml) in order, and then 4 µL of **Boc-Lys-OH** (50 mM in H_2O) was added to these 3 tubes. Next, 4 µL of compd.**4** stock solution (1.0 M in ACN) was added to B and C tubes at room temperature. The reaction mixtures were vortexed for 3 seconds, transferred into heating block pre-heated at37°C, and incubated at 37°C for 2 h. These reactions were analyzed by HPLC and HR-MS following previous method.

4.5 Significance of DTT in pretreatment

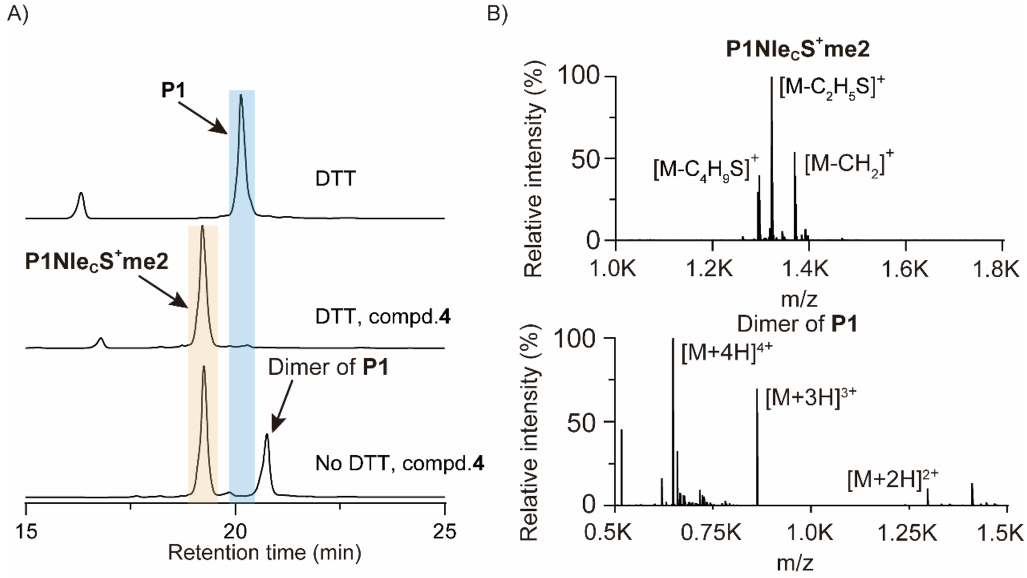

Figure S5 Influence of DTT on the alkylation reaction and analysis by HPLC and HR-MS. (A) The alkylation reactions were analyzed by HPLC after 2h, and the peaks were marked by different colors and labels. (B) HR-MS of the collected peaks were determined by ESI-TOF and the ionic fragments were analyzed and labeled. Dimer of **P1**: $[M+2H]^{2+}$ *m/z* found: 1296.6251, calculated: 1296.7483; $[M+3H]^{3+}$ *m/z* found: 864.5137, calculated: 864.8322; $[M+4H]^{4+}$ *m/z* found: 648.0172, calculated: 648.8842.

At room temperature, 95/91/92 µL of NaPi buffer (4 M GdnHCl, 0.1 M NaPi, pH 7.3) were added to 3 Eppendorf tubes (A/B/C, 1.5 mL), and then 4 µL of **P1** (50 mM in

H_2O) was added. Subsequently, 1/1/0 μL of DTT stock solution (1.0 M in H_2O) were added to these mixtures, respectively. These mixtures were vortexed for 3 seconds, transferred into a heating block pre-heated at 37 °C, and incubated for 1 h. Next, these mixtures were cooled by ice bath for 10 min, and then 0/4/4 μL of compd.**4** stock solution (1.0 M) in NMP were added to these tubes (A/B/C) at room temperature, respectively. These reaction mixtures were vortexed for 3 seconds, transferred into heating block pre-heated at 37°C, and incubated at 37°C for 2 h. These reactions were analyzed following previous method.

4.6 Peptide expansion

At room temperature, 2.0 μL of peptide (50 mM, 1.0 equiv.) in H_2O was added to an Eppendorf tube (1.5 mL) and diluted with 43.5 μL of NaPi buffer (4 M GdnHCl, 0.1 M NaPi, pH 7.3). Subsequently, 2.0 μL of DTT stock solution (0.5 M, 10 equiv.) in H_2O was added to the mixture. The mixture was vortexed for 3 seconds, transferred into a heating block pre-heated at 37 °C, and incubated for 1 h. Next, the mixture was cooled by ice bath for 10 min, and then 2.5 μL of compd.**4** stock solution (1.0 M, 25 equiv.) in NMP was added at room temperature. The reaction mixture was vortexed for 3 seconds, and incubated at 37 °C for 1 h. After finish of this reaction, 2.0 μL of HCl stock solution (6.0 M) was added to quench the reaction. Finally, the sample was prepared by centrifuge at 15000 rpm for 5 min and analyzed by HPLC equipped C18 column (XBridge Peptide BEH C18 column, 130 Å, 5 μm, 4.6 mm × 150 mm). The purified fractions were collected and determined by ESI-TOF.

HPLC analysis conditions and HR-MS of the alkylation reactions:

1) **P2Nle$_C$S$^+$me2 (VREIWQDFKTDANle$_C$S$^+$me2L)**: 10%-50% B in 20 min, the retention time of **P2Nle$_C$S$^+$me2** is at 17.1 min. (ESI-TOF): $[M]^+$ *m/z* found: 1811.90, calculated: 1811.89.

2) **P3Nle$_C$S$^+$me2 (RMNSPKSLHRTGNle$_C$S$^+$me2L)**: 5%-30% B in 20 min, the retention time of **P3Nle$_C$S$^+$me2** is at 12.3 min. HR-MS (ESI-TOF): $[M]^+$ *m/z* found:1687.40, calculated: 1687.86.

3) **P4Nle$_C$S$^+$me2 (KPAIRRLARNle$_C$S$^+$me2GGLIY)**: 10%-50% B in 20 min, the retention time of **P4Nle$_C$S$^+$me2** is at 10.2 min. HR-MS (ESI-TOF): $[M]^+$ *m/z* found: 1774.78, calculated: 1775.05.

5 Preparation of sulfonium peptides in large scale

5.1 Optimization of sulfonium concentrations

To scale up this reaction, reactions at different **P1** concentrations (0/2/5/10 mM) were performed at the optimal condition. The following procedure for these reactions were the same to the previous method.

5.2 Scale up synthesis of sulfonium peptides

Preparation of P1Nle$_C$S$^+$me2

At room temperature, 77.0 μL of **P1** (11.6 mg, 100 mM, 1.0 equiv.) in H_2O was added to an Eppendorf tube (1.5 mL) and diluted with 554.4 μL of NaPi buffer (4 M GdnHCl, 0.1 M NaPi, pH 7.3). Subsequently, 77.0 μL of DTT stock solution (1.0 M, 1.0 equiv.) in H_2O was added to the mixture. The mixture was vortexed for 3 seconds, transferred into a heating block pre-heated at 37 °C, and incubated for 1 h. Next, the mixture was cooled by ice bath for 10 min, and then 61.6 μL of compd.**4** stock solution (1.0 M, 80 mM) in NMP was added at room temperature. The reaction mixture was vortexed for 3 seconds, and incubated at 37°C for 1 h. After finish of this reaction, 10.0 μL of HCl stock solution (6.0 M) was added to quench the reaction. HPLC sample was prepared by centrifuge at 15000 rpm for 5 min, and purification condition is by HPLC equipped with C18 column (XBridge Peptide BEH C18 column, 130 Å, 10 μm, 19 mm × 150 mm) with a gradient of 5%-35% B in 20 min, the retention time of **P1Nle$_C$S$^+$me2** is at 14.6 min. Fractions were collected and determined by ESI-TOF. The target fractions were pooled, evaporated, lyophilized and 11.2 mg of white solid was obtained in 96% yield. The white solid was stored at -20 °C until usage.

6 Preparation of sulfonium peptides

Preparation of H3K4Nle$_C$S$^+$me2

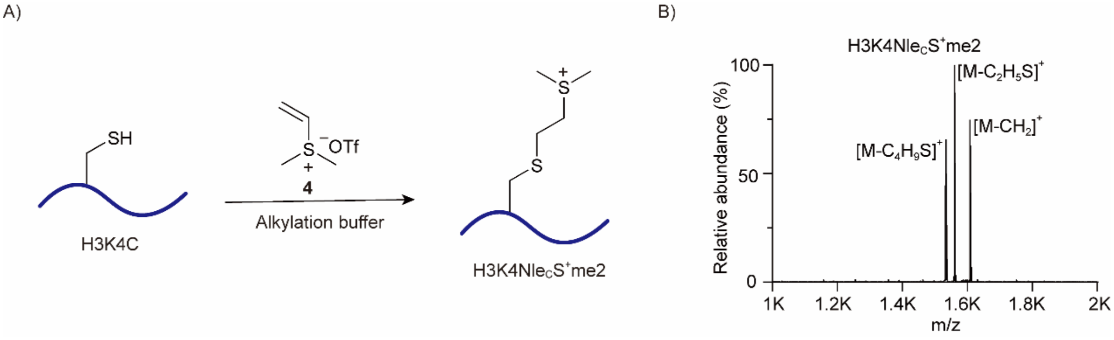

Figure S6 Alkylation of **H3K4C** with compd.**4** and its MS data. (A) **H3K4C** was alkylated with compd.**4** under alkylation buffer. (B) **H3K4Nle$_C$S$^+$me2** was determined with MALDI-TOF: [M-CH_2]$^+$ *m/z* found: 1608.98, calculated: 1608.65; [M-C_2H_5S]$^+$ *m/z* found: 1562.53, calculated: 1562.65; [M-C_4H_9S]$^+$ *m/z* found: 1534.95, calculated: 1534.65.

At room temperature, 130 µL of **H3K4C** (20 mM, 1.0 equiv.) in H_2O was divided into 3 Eppendorf tubes (1.5 ml) equally and 887.6 µL of NaPi buffer (4 M GdnHCl, 0.1 M NaPi, pH 7.3) was added to the tubes, respectively. Subsequently, 43.3 µL of a DTT stock solution (0.5 M, 10 equiv.) in H_2O was added to these three mixtures. These mixtures were vortexed for 3 seconds, transferred into a heating block pre-heated at 37 °C, and incubated for 1 h. Next, these mixtures were cooled by ice bath for 10 min, and 108.3 µL of compd.**4** stock solution (1.0 M, 50 equiv.) in NMP were added to these mixtures at room temperature. These reaction mixtures were vortexed for 3 seconds, and incubated for 2 h. After finish of this reaction, 10.0 µL of a HCl stock solution (6.0 M) was added to quench the reaction. Finally, the sample was prepared by centrifuge at 15000 rpm for 5 min and purified using HPLC equipped C18 column (XBridge Peptide BEH C18 column, 130 Å, 10 µm, 19 mm × 150 mm) with a gradient of 5%-30% B in 20 min, the retention time of **H3K4Nle$_C$S$^+$me2** is at 10.4 min. The fractions were collected and determined by ESI-TOF. The target fractions were pooled, evaporated, lyophilized and 12.5 mg of white solid was obtained in 98% yield. The white solid was stored at -20 °C until usage.

Preparation of H3K9Nle$_C$S$^+$me2

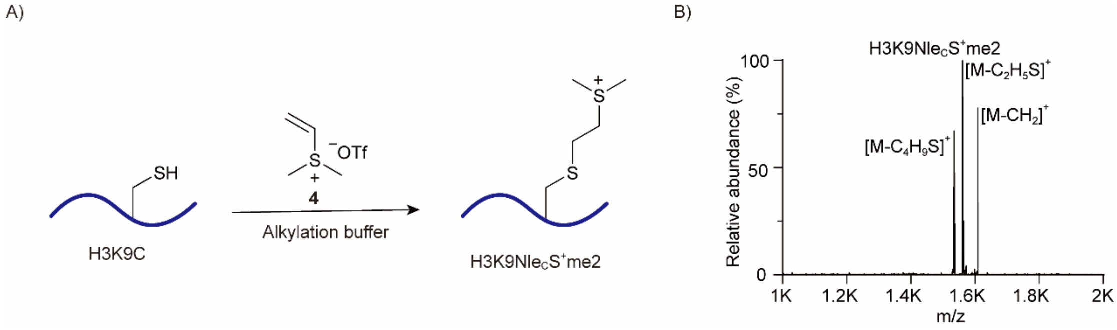

Figure S7 Alkylation of **H3K9C** with compd.**4** and its MS data. (A) **H3K9C** was alkylated with compd.**4** under alkylation buffer. (B) **H3K9Nle$_C$S$^+$me2** was determined with MALDI-TOF: [M-methyl+H]$^+$ *m/z* found: 1608.83, calculated: 1608.65; [M-C_2H_5S]$^+$ *m/z* found: 1562.83, calculated: 1562.65; [M-C_4H_9S]$^+$ *m/z* found: 1534.81, calculated: 1534.65.

The procedure for the preparation of **H3K9Nle$_C$S$^+$me2** was the same as the preparation of **H3K4Nle$_C$S$^+$me2**. The reaction was purified by HPLC equipped C18 column (XBridge Peptide BEH C18 column, 130 Å, 10 µm, 19 mm × 150 mm) with a gradient of 5%-30% B in 20 min, the retention time of **H3K9Nle$_C$S$^+$me2** is at 9.1 min. The fractions were collected and determined by ESI-TOF. The target fractions were pooled, evaporated, lyophilized and 12.2 mg of white solid was obtained in 98% yield. The white solid was stored at -20 °C until usage.

Preparation of H3K9Nle$_C$TMS

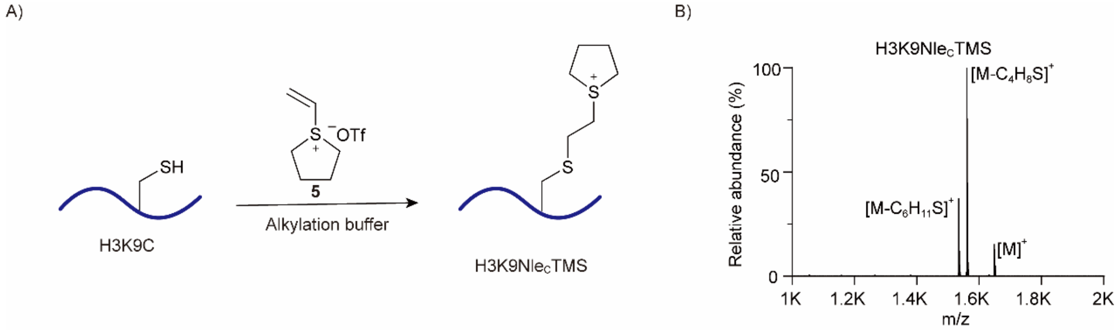

Figure S8 Alkylation of **H3K9C** with compd.**5** and its MS data. (A) **H3K9C** was alkylated with compd.**5** under alkylation buffer. (B) **H3K9Nle$_C$TMS** was determined with MALDI-TOF: [M]$^+$ *m/z* found: 1648.86, calculated: 1648.95; [M-C_4H_8S]$^+$ *m/z* found: 1561.84, calculated: 1561.95; [M-$C_6H_{11}S$]$^+$ *m/z* found: 1534.83, calculated: 1534.95.

The procedure for the preparation of **H3K9Nle$_C$TMS** was the same as the preparation of **H3K4Nle$_C$S$^+$me2**. The reaction was purified by HPLC equipped C18 column (XBridge Peptide BEH C18 column, 130 Å, 10 μm, 19 mm × 150 mm) with a gradient of 5%-30% B in 20 min, the retention time of **H3K9Nle$_C$TMS** is at 9.8 min. The fractions were collected and determined by ESI-TOF. The target fractions were pooled, evaporated, lyophilized and 12.5 mg of white solid was obtained in 97% yield. The white solid was stored at -20 °C until usage.

Preparation of H3K9Nle$_C$PMS

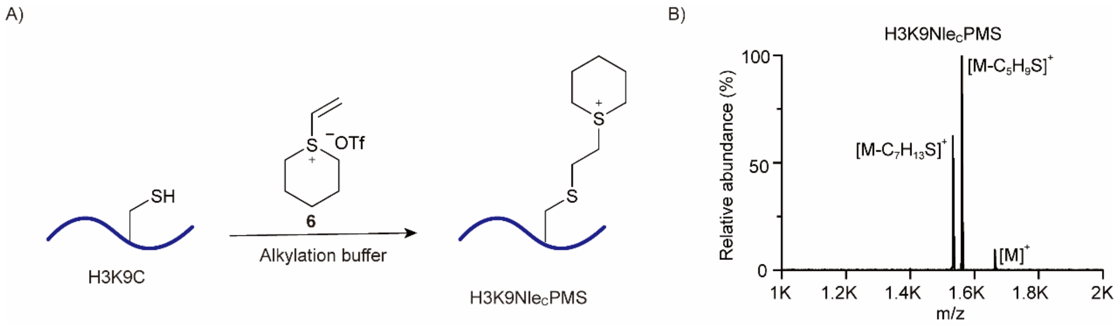

Figure S9 Alkylation of **H3K9C** with compd.**6** and its MS data. (A) **H3K9C** was alkylated with compd.**6** under alkylation buffer. (B) **H3K9Nle$_C$PMS** was determined with MALDI-TOF: $[M]^+$ *m/z* found: 1663.92, calculated: 1663.98; $[M\text{-}C_5H_9S]^+$ *m/z* found: 1561.87, calculated: 1561.78; $[M\text{-}C_7H_{13}S]^+$ *m/z* found: 1534.84, calculated: 1534.48.

The procedure for the preparation of **H3K9Nle$_C$PMS** was the same as the preparation of **H3K4Nle$_C$S$^+$me2**. The reaction was purified by HPLC equipped C18 column (XBridge Peptide BEH C18 column, 130 Å, 10 μm, 19 mm × 150 mm) with a gradient of 5%-30% B in 20 min, the retention time of **H3K9Nle$_C$PMS** is at 11.4 min. The fractions were collected and determined by ESI-TOF. The target fractions were pooled, evaporated, lyophilized and 2.0 mg of white solid was obtained in 19% yield. The white solid was stored at -20 °C until usage.

Preparation of H3K4NleS⁺me2

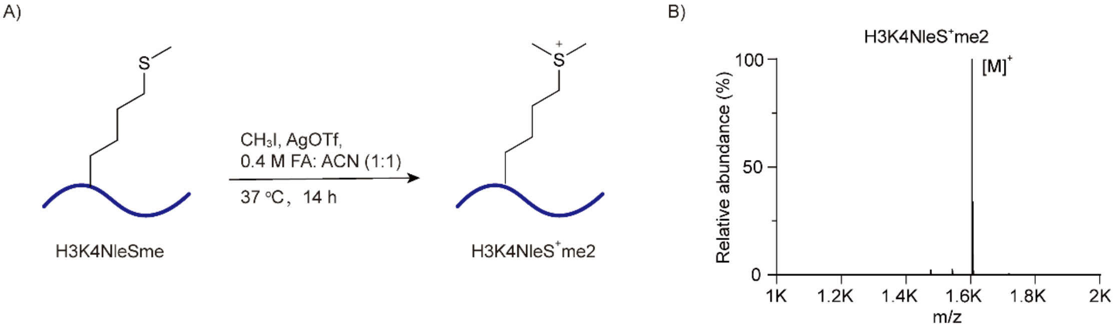

Figure S10 Alkylation of **H3K4NleSme** with CH_3I and its MS data. (A) **H3K4NleSme** was alkylated with CH_3I under alkylation buffer. (B) **H3K4NleS$^+$me2** was determined with ESI-TOF: $[M]^+$ *m/z* found: 1604.84, calculated: 1604.88.

At room temperature, 73.0 µL of H3K4NleSme (100 mM, 1.0 equiv.) in H_2O was divided into 4 Eppendorf tubes (1.5 ml) equally and diluted with 933.8 µL of combined solvent (0.4 M FA: ACN, 1:1) respectively. Subsequently, 41.5 µL of a CH_3I stock solution (1.0 M, 20 equiv.) in ACN and 41.5 µL of AgOTf stock solution (1.0 M, 20 equiv.) in H_2O were added to these four mixtures. The reaction mixtures were vortexed, transferred into a Thermocycler pre-heated at 37 °C, and incubated at 800 rpm for 16 h. After finish of these reactions, grey precipitant formed and was removed by centrifuge at 15000 rpm for 5 min. The supernatants were pooled and the solvent was removed under vacuum condition. The solid crude was redissolved in 3 mL of acetone followed by centrifuge at 15000 rpm for 5 min. The crude was purified by HPLC and the purified condition is using HPLC equipped C18 column (XBridge Peptide BEH C18 column, 130 Å, 10 µm, 19 mm × 150 mm) with a gradient of 5%-30% B in 20 min, the retention time of **H3K9NleS$^+$me2** is at 8.6 min. Fractions were collected and determined by ESI-TOF. The target fractions were pooled, evaporated, lyophilized and 3.6 mg of white solid was obtained in 36% yield. The white solid was stored at -20 °C until usage.

Preparation of H3K9NleS⁺me2

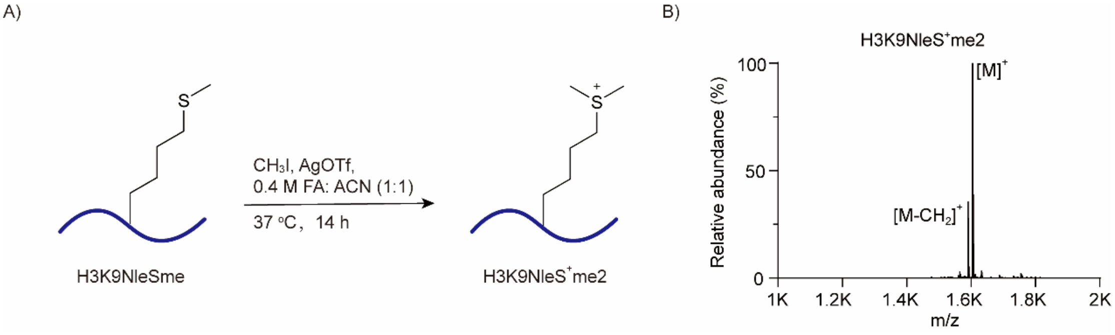

Figure S11 Alkylation of **H3K9NleSme** with compound CH_3I and its MS data. (A) **H3K9NleSme** was alkylated with CH_3I under alkylation buffer. (B) **H3K9NleS⁺me2** was determined with ESI-TOF: $[M]^+$ *m/z* found: 1604.74, calculated: 1604.88; $[M\text{-}CH_2]^+$ *m/z* found:1590.83, calculated: 1590.85.

The procedure to prepare **H3K9NleS⁺me2** was the same as the preparation of **H3K4NleS⁺me2**. The crude was purified by HPLC and the purified condition is using HPLC equipped C18 column (XBridge Peptide BEH C18 column, 130 Å, 10 µm, 19 mm × 150 mm) with a gradient of 5%-30% B in 20 min, the retention time of **H3K9NleS⁺me2** is at 11.7 min. Fractions were collected and determined by MALDI-TOF. The target fractions were pooled, evaporated, lyophilized and 9.7 mg of white solid was obtained in 96% yield. The white solid was stored at -20 °C until usage.

Preparation of compound 7

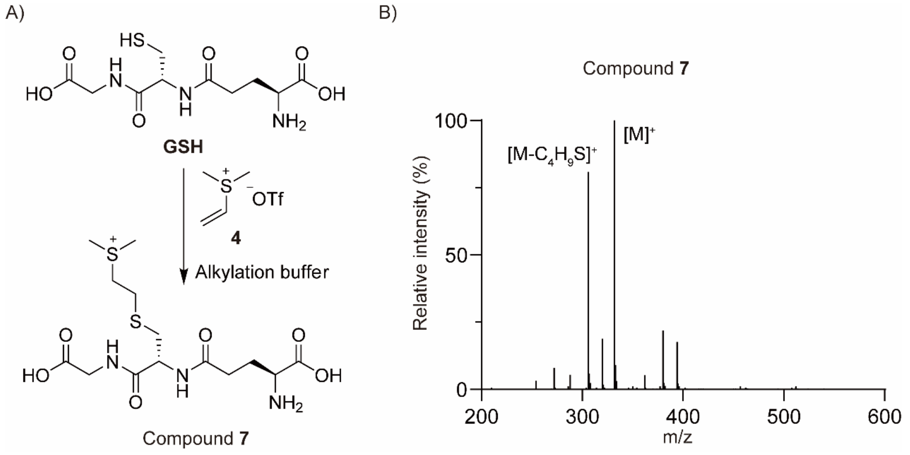

Figure S12 Alkylation of **GSH** with compound **4** and its MS data. (A) **GSH** was alkylated with

compound **4** under alkylation buffer to obtain compound **7**. (B) Compound **7** was determined with HR-MS: $[M]^+$ *m/z* found: 396.0779, calculated: 396.1258; $[M-C_4H_9S]^+$ *m/z* found: 307.0519, calculated: 307.0760.

Procedure for the preparation of compound **7** was the same as the preparation of **H3K4Nle$_C$S$^+$me2**. The reaction was purified by HPLC equipped C18 column with a gradient of 0-5% ACN in 20 min, the retention time of compound **7** is at 5.8 min. The fractions were collected and determined by ESI-TOF. The target fractions were pooled, evaporated, lyophilized and 9.6 mg of white solid was obtained in 95% yield. The white solid was stored at -20 °C until usage.

Preparation of compound 8

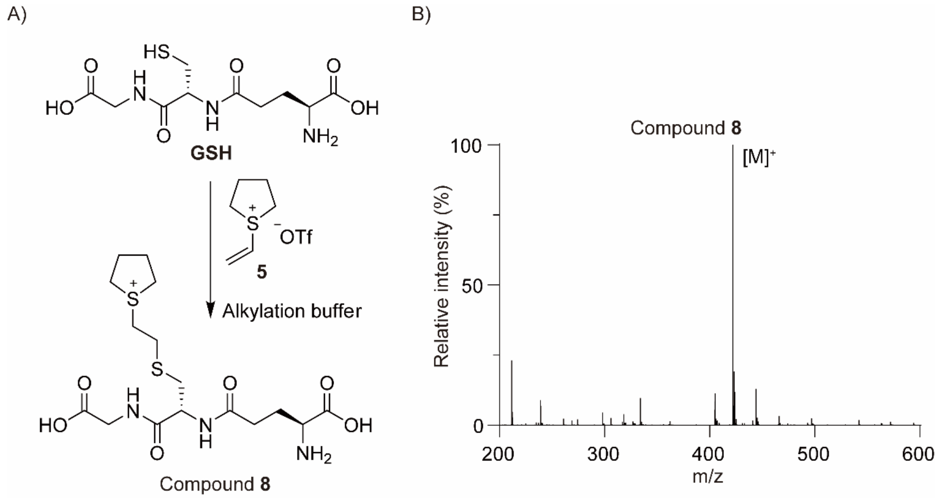

Figure S13 Alkylation of **GSH** with compound **5** and its MS data. (A) **GSH** was alkylated with compound **5** under alkylation buffer to obtain compound **8**. (B) Compound **8** was determined with HR-MS: $[M]^+$ *m/z* found: 424.1403, calculated: 424.1414.

Procedure for the preparation of compound **8** was the same as the preparation of **H3K4Nle$_C$S$^+$me2**. The reaction was purified by HPLC equipped C18 column with a gradient of 0-5% ACN in 20 min, the retention time of compound **8** is at 11.3 min. The fractions were collected and determined by ESI-TOF. The target fractions were pooled, evaporated, lyophilized and 14.5 mg of white solid was obtained in 96% yield. The white solid was stored at -20 °C until usage.

Preparation of H3K4Nle$_C$S$^+$me2K16bio

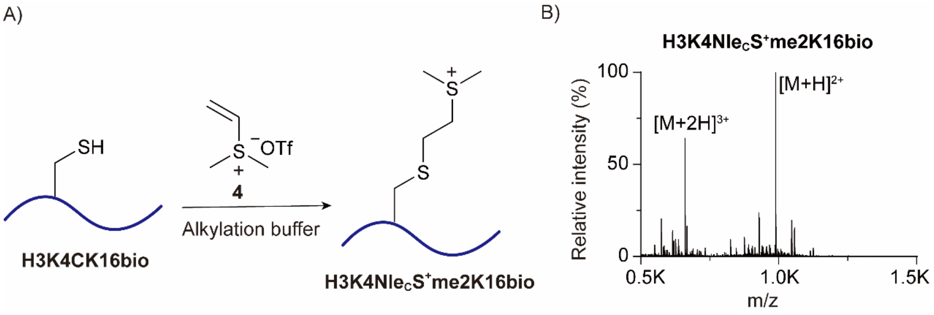

Figure S14 Alkylation of **H3K4CK16bio** with compound **4** and its MS data. (A) **H3K4CK16bio** was alkylated with compound **4** under alkylation buffer to obtain **H3K4Nle$_C$S$^+$me2K16bio**. (B) **H3K4Nle$_C$S$^+$me2K16bio** was determined with HR-MS: $[M+H]^{2+}$ *m/z* found: 989.4326, calculated: 989.6310; $[M+2H]^{3+}$ *m/z* found: 660.0172, calculated: 660.0873.

Procedure for the preparation of **H3K4Nle$_C$S$^+$me2K16bio** was the same as the preparation of **H3K4Nle$_C$S$^+$me2**. The reaction was purified by HPLC equipped C18 column with a gradient of 5%-30% ACN in 20 min, the retention time of **H3K4Nle$_C$S$^+$me2K16bio** is at 17.8 min. The fractions were collected and determined by ESI-TOF. The target fractions were pooled, evaporated, lyophilized and 15.6 mg of white solid was obtained in 98% yield. The white solid was stored at-20 °C until usage.

7 General procedure of crosslinking between sulfonium probes and readers

Sulfonium peptide was added followed by reader protein to crosslink buffer (0.1 M HEPES, pH 7.5). The mixture (total volume: 30 μL) was mixed by pipette softly, transferred into a 96-well plate, and incubated on ice for 15 min. Next, the mixture was exposed to 302 nm ultraviolet irradiation with 305 nm optical filter in an ice bath. The energy was set as 7200 × 100 μJ/cm^2. The reaction mixture was later analyzed by LC-MS to calculate analytical yield.

Determination of the yield of the crosslinking reaction between sulfonium probes and reader proteins was either achieved via peak integration after PDA analysis at 220 nm or based on peak areas of reconstructed zero-charge spectra after deconvolution. PDA analysis was used in the cases where protein peak separation was achieved via HPLC, but the peak of our crosslinking reaction could not be separated, so the yield was determined via peak areas obtained from the zero-charge spectra after deconvolution. In the case of zero-charge analysis, only product peaks resulting from the major peak present in the starting material were considered. Minor peak reactivity was reported but not considered for yield determination because side reactivities might go undetected due to the lower overall signal intensity. The determined yields should be considered semi-quantitative due to unknown extinction coefficients and altered ionization properties of newly formed species. The yield was calculated as follows:

$$Yield = \frac{peak\ area\ of\ the\ desired\ product}{sum\ of\ peak\ areas\ of\ crosslink\ products\ and\ the\ starting\ reader\ proteins} \times 100\%$$

7.1 Crosslink CBX1, MPP8, BPTF with sulfonium peptides

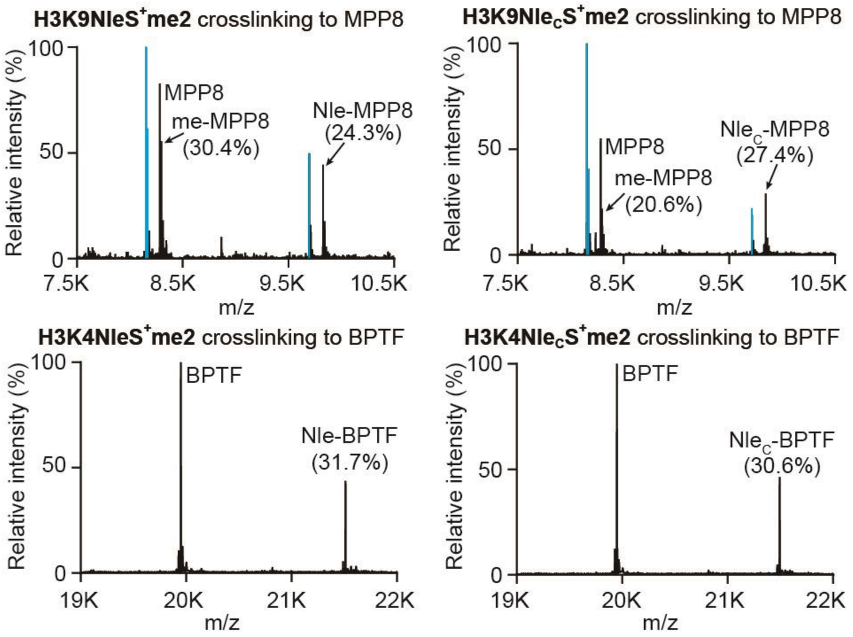

Figure S15 Comparison of reader crosslinking activities between NleS$^+$me2 and Nle$_C$S$^+$me2. HR-MS of crosslinking mixtures of NleS$^+$me2 or Nle$_C$S$^+$me2 and MPP8 or BPTF (15 min). The yields were quantified from mass peak integrations. The recombinant MPP8 also contains a proteoform with loss of *N*-methionine that was labelled as cyan peaks.

A mixture of 100 μM sulfonium peptide (H3K9NleS$^+$me2 or H3K9Nle$_C$S$^+$me2 or H3K4NleS$^+$me2 or H3K4Nle$_C$S$^+$me2) and 5 μM reader protein (MPP8 or BPTF) was crosslinked for 15 min by general procedure. The analytical yield was calculated as the method described above.

7.2 Crosslink CBX1, MPP8 with H3K9NleS$^+$me2, H3K9Nle$_C$S$^+$me2, H3K9Nle$_C$TMS

A mixture of 100 μM sulfonium peptide (H3K9NleS$^+$me2 or H3K9Nle$_C$S$^+$me2 or H3K9Nle$_C$TMS) and 3 μM reader protein (CBX1 or MPP8) was crosslinked for 5 min (for CBX1) or 20 min (for MPP8) by general procedure. The analytical yield was calculated as the method described above.

7.3 Crosslink CBX1, MPP8 with H3K9Nle$_C$TMS, H3K9Nle$_C$PMS

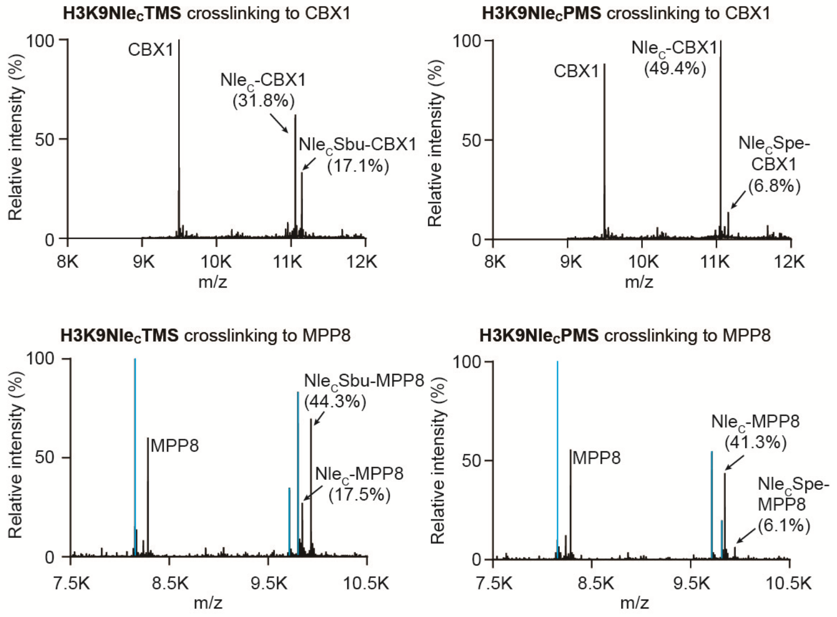

Figure S16 Analysis of reader crosslinking activities between cyclic sulfonium probes (Nle$_C$TMS or Nle$_C$PMS) and readers. HR-MS of the reaction mixtures of **H3K9Nle$_C$TMS** or **H3K9Nle$_C$PMS** and CBX1 or MPP8 under UV-B irradiation for 15 min. The yields were quantified by mass peak integrations. The recombinant MPP8 also contains a proteoform with loss of *N*-methionine that was labelled as cyan peaks.

A mixture of 100 µM sulfonium peptide (H3K9Nle$_C$TMS or H3K9Nle$_C$PMS) and 5 µM reader protein (CBX1 or MPP8) was crosslinked for 15 min by the general procedure. The analytical yield was calculated as the method described above.

8 Crosslinking kinetics comparison between sulfonium probes and readers

Different concentrations of sulfonium probes crosslink with the corresponding reader protein by the general crosslink procedure. The reaction was analyzed by LC-MS and yield was calculated. To study the kinetics of the crosslinking reaction between sulfonium probes and reader proteins, we need to calculate the K_m and k_{cat} of the reaction by the Michaelis-Menten equation. Michaelis-Menten equation is: $v_0 = (Vmax*[S])/ (K_m+[S])$, so our prior work is to calculate the v_0 of the crosslinking reaction. Our previous kinetics investigation of the crosslinking reaction has demonstrated that the crosslinking reaction rate at different concentrations of sulfonium peptides remained unchanged within 3 minutes, which was approximately equal to v_0. Thus, we extracted peak areas obtained from the zero-charge spectra after deconvolution, calculated the peptidyl labeling yield within 3 minutes of the crosslinking reaction, and further calculated the reaction rate at different concentrations of sulfonium probe. Finally, the Michaelis-Menten equation curve was drawn by GraphPad software, and the K_m and k_{cat} of the reaction were calculated by this software.

8.1 Kinetics study of the crosslinking between sulfonium probes and CBX1

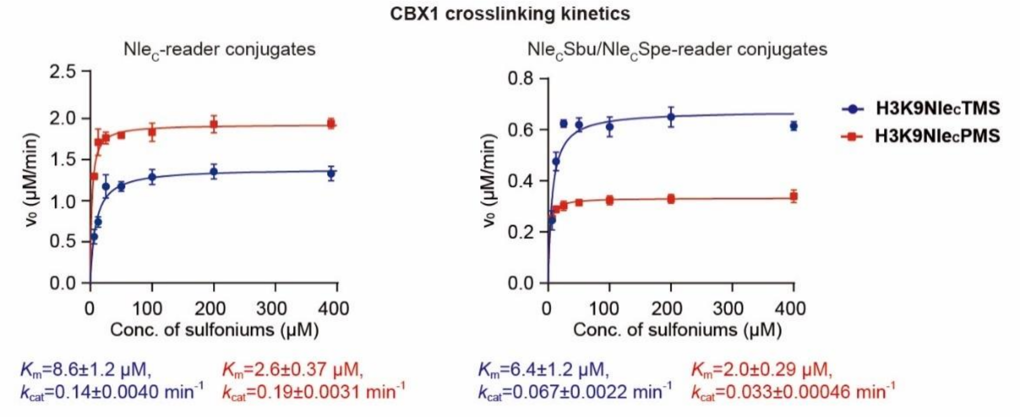

Figure S17 Kinetic curves of crosslinking between sulfonium probes and CBX1. 10 μM CBX1 was treated with **H3K9Nle$_C$TMS** or **H3K9Nle$_C$PMS** at various concentrations for 3 min photo crosslinking. The products were quantified by mass spectrometry and the calculated initial reaction rates v_0 were used for the plot with peptide concentrations by Michaelis-Menten equation, error bars represent mean ± SE (n = 3 crosslinking replicates).

Parallel experiments containing 7 groups were performed as the following to investigate the kinetics of the crosslinking between sulfonium probes and CBX1.

At room temperature, sulfonium probes in H_2O were added to 7 Eppendorf tubes (1.5 mL) and diluted the concentrations of sulfonium probe with HEPES buffer (0.1 M HEPES, pH 7.5) to be at 6.25/12.5/25/50/100/200/400 μM, respectively. Subsequently, CBX1 stock solution was added to the mixture to be at 10.0 μM. These mixtures were crosslinked for 3 min by general procedure. The analytical yield and kinetic parameters were calculated as the method described above.

8.2 Kinetics study of the crosslinking between sulfonium probes and BPTF

At room temperature, sulfonium probes in H_2O were added to 7 Eppendorf tubes (1.5 mL) and diluted the concentrations of sulfonium probe with HEPES buffer (0.1 M HEPES, pH 7.5) to be at 1.56/3.13/6.25/12.5/25/50/100 μM, respectively. Subsequently, BPTF stock solution was added to the mixture to be at 5.0 μM. The following procedure for the kinetics investigation of the reaction between sulfonium peptide and BPTF was the same to that of CBX1.

9 Proteome-wide crosslinking experiment

HeLa cells obtained from ATCC were cultured in Dulbecco's modified Eagle's medium (DMEM, Gibco, C11995500BT) supplemented with 10% fetal bovine serum (FBS, CellMax, SA211.02), 100 U/mL penicillin, and 100 μg/mL streptomycin. Cells were maintained in a humidified 37 °C incubator with 5% CO_2.

The proteomic experiment was carried out as reported previously with several differences.[1] Approximately 1.8×10^7 HeLa cells were harvested, washed with chilled PBS, and lysed in hypotonic buffer (10 mM Tris, 15 mM NaCl, 1.5 mM $MgCl_2$, 0.2 mM PMSF, pH 7.5). The suspension was incubated on ice for 10~15 min and mixed by inverting up and down regularly. After centrifugation at 200 g for 5 min, the pellet was resuspended in hypotonic buffer and homogenized with three strokes of a loose pestle Dounce homogenizer. The intact nuclei were collected by spinning at 200 g for 5 min. 50 μM sulfonium probes (H3K4NleS$^+$me2(1-15)-K(desthiobiotin) or H3K4Nle$_C$S$^+$me2K16bio) and 500 μM unmodified peptide H3 (1-15) were added to nuclei which were resuspended by crosslink buffer (100 mM HEPES, 150 mM KCl, 1.5 mM $MgCl_2$, 1 mM PMSF, pH 7.5) in group A. The same amount of sulfonium probes and competitive peptide H3K4me3 (1-15) were added in group B. H3K4NleS$^+$me2(1-15)-K(desthiobiotin), H3(1-15), H3K4me3(1-15) were from the previous work[1]. The mixture was transferred to a 24-well plate, incubated on ice for 10 min, and irradiated under 302 nm ultraviolet light with 305 nm filter for 5 min on ice. The energy was set as 7200×100 μJ/cm^2. Subsequently, the nuclei were washed by crosslink buffer twice to remove excess sulfonium probes. Next, the nuclei were resuspended by nuclear protein extraction buffer (50 mM HEPES, 300 mM KCl, 1.5 mM $MgCl_2$, 0.1% NP40, 10% glycerol, 0.2 mM PMSF, pH 7.5) and sonicated to obtain nuclear protein. After centrifugation at 13000 rpm for 5 min, the supernatant was loaded to Pierce streptavidin magnetic beads (Thermo Fisher, 88817), which were equilibrated and blocked with nuclear protein extraction buffer containing 2% BSA.

After rotation at 4 °C for 3 hours, the beads were washed by wash buffer (50 mM

HEPES, 150 mM KCl, 1.5 mM $MgCl_2$, 5% glycerol, pH 7.5) twice and 50 mM ammonium bicarbonate (NH_4HCO_3) twice. Subsequently, the samples were treated with 10 mM dithiothreitol (DTT) in 50 mM NH_4HCO_3 at 37 ºC at 1000 rpm in a mixer for 1 h followed by treatment with 20 mM iodoacetamide (IAA) at room temperature at 1000 rpm for 30 min in dark. After washing the beads with NH_4HCO_3 for three times, Trypsin Gold (2 μL of 1 μg/μL stock) was added to the beads in 200 μL 50 mM NH_4HCO_3 to perform on-bead digestion at 37 ºC at 1500 rpm for 12 h in a mixer. Extra 1 μL trypsin was added for another 2 h digestion. The supernatant was collected, and the beads were washed with another 200 μL 50 mM NH_4HCO_3. The combined supernatants were centrifuged and lyophilized for further LC-MS/MS analysis.

For LC-MS/MS analysis, the peptides were separated by a 153 min gradient elution at a flow rate 0.300 μL/min with the Thermo EASY-nLC1200 integrated nano-HPLC system which is directly interfaced with the Thermo Orbitrap Exploris 480 mass spectrometer. The analytical column was a home-made fused silica capillary column (75 μm inner diameter, 150 mm length; Upchurch, Oak Harbor, WA) packed with C18 resin (300 Å, 3 μm, Varian, Lexington, MA). Mobile phase A consisted of 0.1% formic acid, and mobile phase B consisted of 80% acetonitrile and 0.1% formic acid. The mass spectrometer was operated in a FAIMS (CV, -45, -65) data-dependent acquisition mode using the Xcalibur 4.5 software, and there is a single full-scan mass spectrum in the Orbitrap (350-1800 m/z, 60,000 resolution) followed by MS/MS scans (15,000 resolution) for 1s of cycle time at 30% normalized collision energy.

Each mass spectrum was analyzed using Proteome Discoverer 2.5 for the database searching against the Homo sapiens (Human) proteome database downloaded from UniProtKB (UP000005640). The Sequest search parameters included a 10 ppm precursor mass tolerance, 0.02 Da fragment ion tolerance, and up to 2 internal cleavage sites. Fixed modifications included cysteine alkylation, and methionine oxidation was variable modification. All data were filtered at 1% false discovery rate (FDR) at peptide spectrum match (PSM)/Precursor, Peptide, and Protein.

10 Preparation of sulfonium proteins

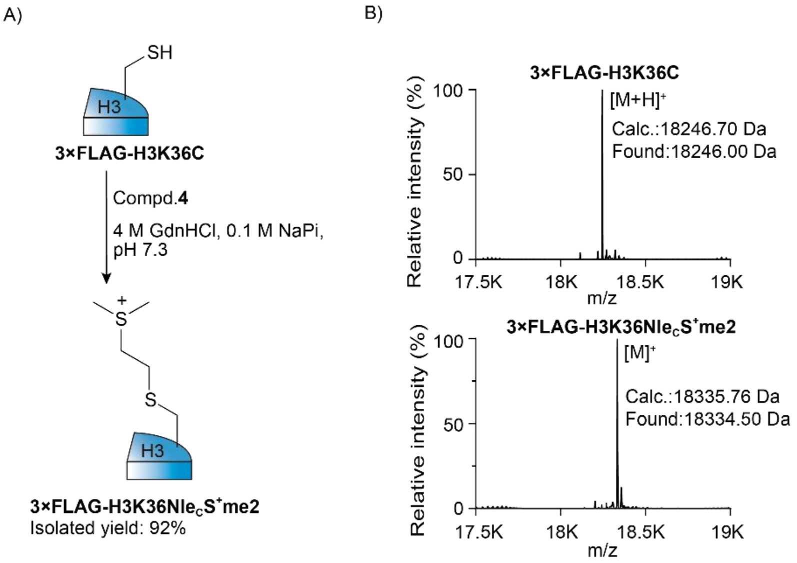

Figure S18 Alkylation of **3×FLAG-H3K36C** with compd.**4** and its MS data. A) **3×FLAG-H3K36C** was alkylated with compd.**4** under alkylation buffer. B) **3×FLAG-H3K36Nle$_C$S$^+$me2** was determined with ESI-TOF: $[M]^+$ *m/z* found: 18334.50, calculated: 18335.76.

At room temperature, 66 μL of **3×FLAG-H3K36C** (1 mM, 1.0 equiv.) in NaPi buffer (4 M GdnHCl, 0.1 M NaPi, pH 7.3) was added to Eppendorf tube (1.5 mL) equally and diluted with 60 μL of NaPi buffer. Subsequently, 2.6 μL of a DTT stock solution (0.5 M, 20 equiv.) in H_2O was added to the mixture. The mixture was vortexed for 3 seconds, transferred into a heating block pre-heated at 37 °C, and incubated for 1 h. Next, these mixtures were cooled by ice bath for 10 min, and then 3.4 μL of compd.**4** stock solution (1.0 M, 50 equiv.) in NMP were added to these mixtures at room temperature. The reaction mixture was vortexed for 3 seconds, and incubated for 1 h. After finish of the reaction, 2.0 μL of a HCl stock solution (6.0 M) was added to quench the reaction. Finally, sample was prepared by centrifuge at 15000 rpm for 5 min and purified by HPLC. The purified condition is using HPLC equipped C4 column (XBridge Protein BEH C4 column, 300 Å, 5 μm, 10 mm × 250 mm) with a gradient of 20%-70% B in 25 min, the retention time of **3×FLAG-H3K36Nle$_C$S$^+$me2** is at 21.5 min. Fractions were collected and determined by ESI-TOF. The target fractions were pooled, evaporated, lyophilized and 1.1 mg of white solid was obtained in 92% yield.

The white solid was stored at -80 °C until usage.

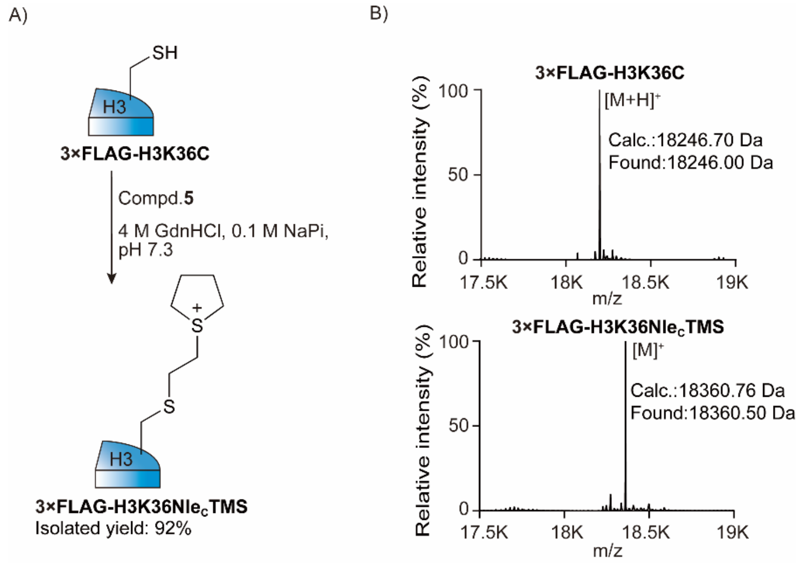

Figure S19 Alkylation of **3×FLAG-H3K36C** with compd.**5** and its MS data. A) **3×FLAG-H3K36C** was alkylated with compd.**5** under alkylation buffer. B) **3×FLAG-H3K36Nle$_{C}$TMS** was determined with ESI-TOF: $[M]^{+}$ *m/z* found: 18360.50, calculated: 18360.76.

The procedure for the preparation of **3×FLAG-H3K36Nle$_{C}$TMS** was the same to the preparation of **3×FLAG-H3K36Nle$_{C}$S^{+}me2**. The reaction was purified by HPLC equipped C4 column (XBridge Protein BEH C4 column, 300 Å, 5 µm, 10 mm × 250 mm) with a gradient of 20%-70% B in 25 min, the retention time of **3×FLAG-H3K36Nle$_{C}$TMS** is at 21.4 min. Fractions were collected and determined by ESI-TOF. The target fractions were pooled, evaporated, lyophilized and 1.1 mg of white solid was obtained in 92% yield. The white solid was stored at -80 °C until usage.

11 Preparation of sulfonium nucleosomes

Preparation of core histones and Widom 601 DNA

3×FLAG tag was cloned into the H3 sequence. H3K36C, H2AK119C mutation was introduced into 3×FLAG-H3 or H2A sequence by site-directed mutagenesis. The *X. laevis* core histones H2A, H2B, H3, H4, H2AK119C, 3×FLAG-H3K36C, and Widom 601 DNA were prepared as reported.[2] 3xFLAG-H3K36C protein was alkylated to form 3xFLAG-H3K$_C$36me3 as described previously.[3]

Preparation of fluorophore-labeled H2A

H2AK119C was alkylated by Cy5-maleimide to introduce fluorescent groups for MST experiments. Purified H2AK119C (final concentration: 0.72 mM) was dissolved in alkylation buffer (1 M HEPES, 4 M Gdn·HCl, 20 mM TCEP, 10 mM L-methionine, pH 7.8) and incubated at 37 ºC for 1 h. The tubes were cooled down on ice for more than 10 min. 4 equivalents of Cy5-maleimide (50 mM dissolved in ddH$_2$O) was added and reacted at 4 ºC for 12 h. 2 extra equivalents of Cy5-maleimide was added for another 8 h reaction at 4 ºC. The reaction was quenched by excess *β*-mercaptoethanol (BME) and the alkylated protein was purified by C4 column (XBridge Protein BEH C4 column, 300 Å, 5 µm, 10 mm × 250 mm) with a gradient of 30%-80% B. The product was confirmed by MALDI-TOF MS and target fractions were evaporated, lyophilized, and stored at -80 ºC.

Refolding of histone octamers and reconstitution of nucleosomes

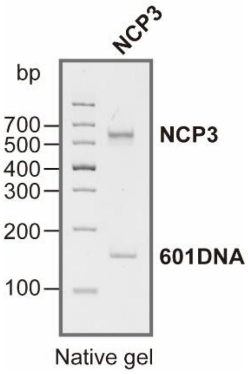

Figure S20 Native gel of **NCP3**.

For different nucleosomes, corresponding histones were dissolved in unfolding buffer (20 mM Tris, 6 M Gdn·HCl, 5 mM BME, pH 7.5). It should be noted that no DTT but BME was used in the whole process when preparing nucleosomes containing sulfonium groups. H2A, H2B, H3, and H4 were mixed with a molar ratio of 1.2:1.2:1:1 and the mixture dialyzed against refolding buffer (10 mM Tris, 2 M NaCl, 5 mM BME, 1 mM EDTA, pH 7.5) for three times, each time for at least 4 h. Octamers were purified by Superdex 200 Increase 10/300 GL column and concentrated.

Octamer and 601 DNA were mixed in reconstitution buffer (10 mM Tris, 2 M KCl, 1 mM EDTA, 2 mM BME, pH 7.5), transferred to a dialysis cassette and ran the gradient dialysis from RB-high buffer (20 mM Tris, 2 M KCl, 1 mM EDTA, 2 mM BME, pH 7.5) to RB-low buffer (10 mM Tris, 250 mM KCl, 1 mM EDTA, 2 mM BME, pH 7.5) for 40 h by a peristaltic pump in the cold room. The mixture dialyzed against TCS buffer (20 mM Tris, 200 mM KCl, 0.5 mM EDTA, 2 mM BME, pH 7.5) for another 3 h and the clarified mixture was injected to HPLC with a TSK gel DEAE-5PW anion exchange column and purified with a gradient of 20%-100% B. Buffer A was TES250 (10 mM Tris, 250 mM KCl, 0.5 mM EDTA, pH 7.5), and buffer B was TES600 (10 mM Tris, 600 mM KCl, 0.5 mM EDTA, pH 7.5). HE buffer was added to each fraction immediately to dilute the concentration of KCl. The target fractions were collected, concentrated, and dialyzed against HE buffer (20 mM HEPES, 1 mM EDTA, 2 mM BME, pH 7.5) for 3 times. The final nucleosome was characterized by SDS-PAGE and native-PAGE and stored at 4 °C.

12 Stability study of sulfonium peptides and nucleosomes

12.1 Stability study of sulfonium peptides

At room temperature, 10 mg of compound **7** was divided into two parts and dissolved in 550 μL of phosphate buffer (0.1 M in D_2O, pH 7.3) and 550 μL of carbonate buffer (0.1 M in D_2O, pH 9.5), respectively. These 2 mixtures were transferred into nuclear magnetic tubes at room temperature and monitored by nuclear magnetic resonance (^{1}H-NMR) at different time points (0 h, 4 h, 8 h, 24 h, 48 h). To obtain more information about the stability of the sulfoniums at pH 7.3 and 9.5, 10 μL of sulfoniums in deuterium buffer were collected from nuclear magnetic tubes at 24 h and 48 h time points. These 2 sulfonium mixtures were diluted with 100 μL of ddH_2O and analyzed by HPLC equipped C18 column with a gradient of 0-5% ACN in 20 min.

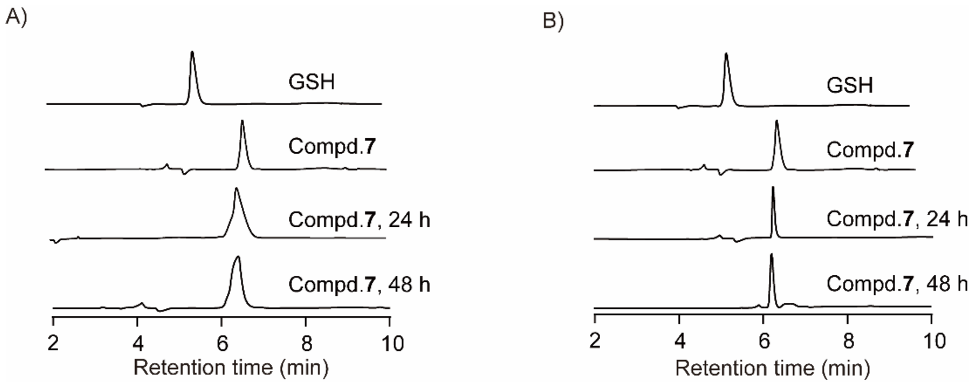

Figure S21 Stability of compound **7** in phosphate buffer analyzed by HPLC. (A) Compound **7** in 0.1 M phosphate buffer (pH 7.3) was monitored by HPLC at 24 and 48 h time points. (B) Compound **7** in 0.1 M carbonate buffer (pH 9.5) was monitored by HPLC at 24 and 48 h time points.

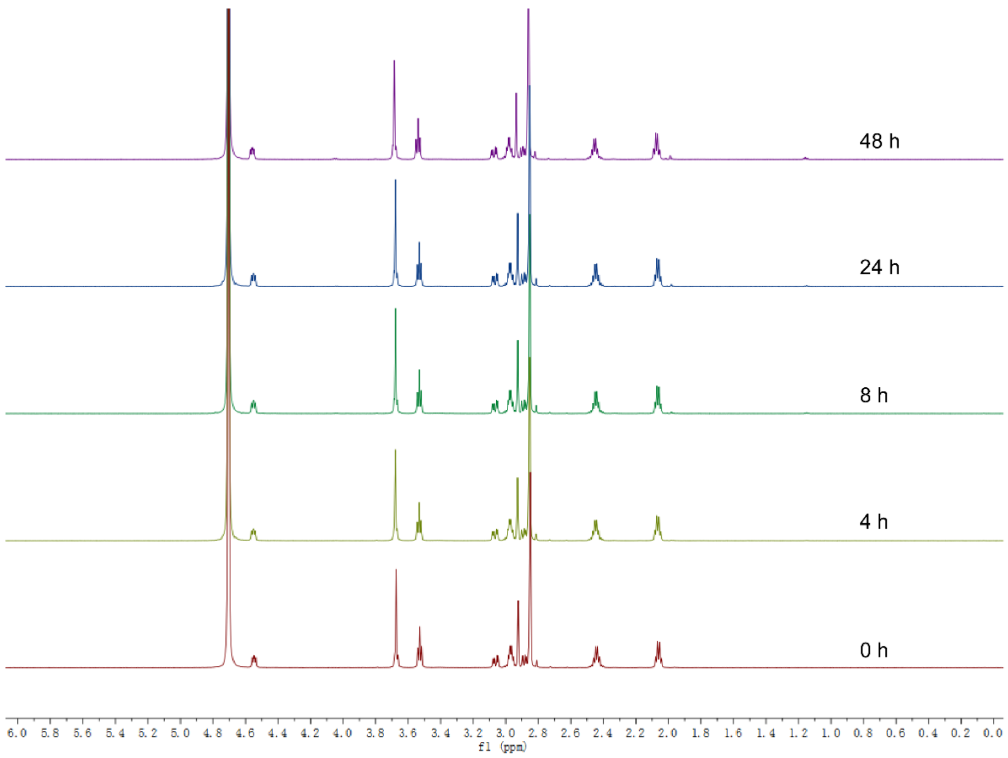

Figure S22 ^{1}H-NMR of compound **7** in phosphate buffer (pH 7.3) at different time points (0/4/8/24/48 h).

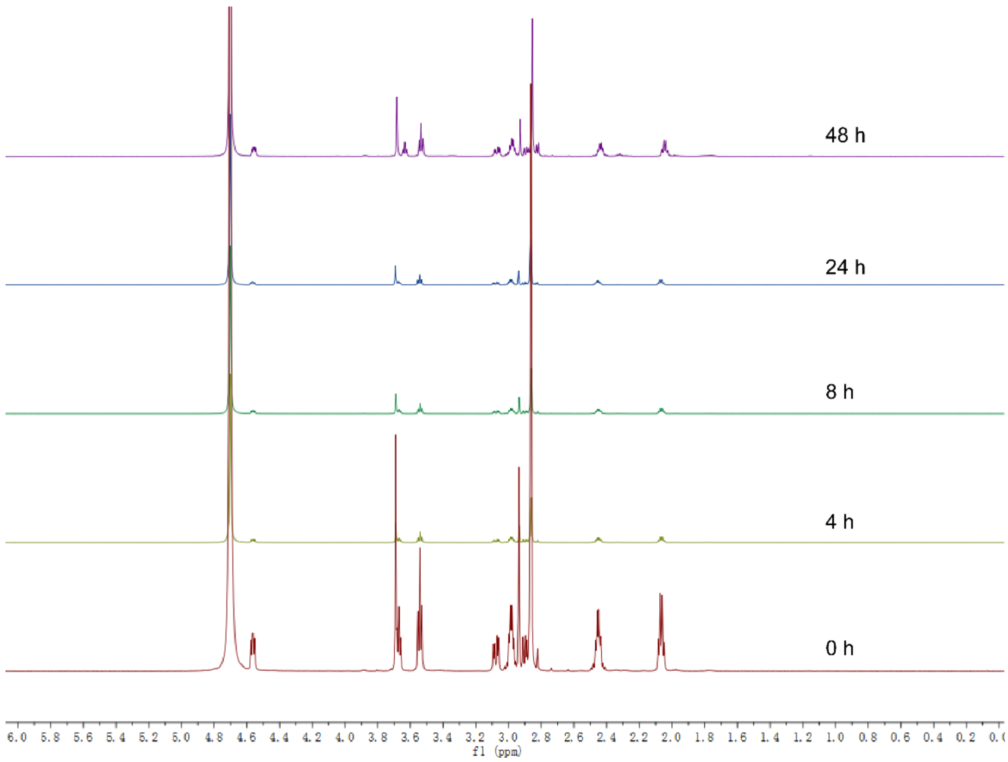

Figure S23 ^{1}H-NMR of compound **7** in carbonate buffer (pH 9.5) at different time points (0/4/8/24/48 h).

At room temperature, 10 mg of compound **8** was divided into two parts and dissolved in 550 μL of phosphate buffer (0.1 M in D_2O, pH 7.3) and 550 μL of carbonate buffer (0.1 M in D_2O, pH 9.5), respectively. These 2 mixtures were transferred into nuclear magnetic tubes at room temperature and monitored by nuclear magnetic resonance (^{1}H-NMR) at different time points (0 h, 4 h, 8 h, 24 h, 48 h). To obtain more information about the stability of the sulfoniums at pH 7.3 and 9.5, 10 μL of sulfoniums in deuterium buffer were collected from nuclear magnetic tubes at 24 h and 48 h time points. These 2 sulfonium mixtures were diluted with 100 μL of ddH_2O and analyzed by HPLC equipped C18 column with a gradient of 0-5% ACN in 20 min.

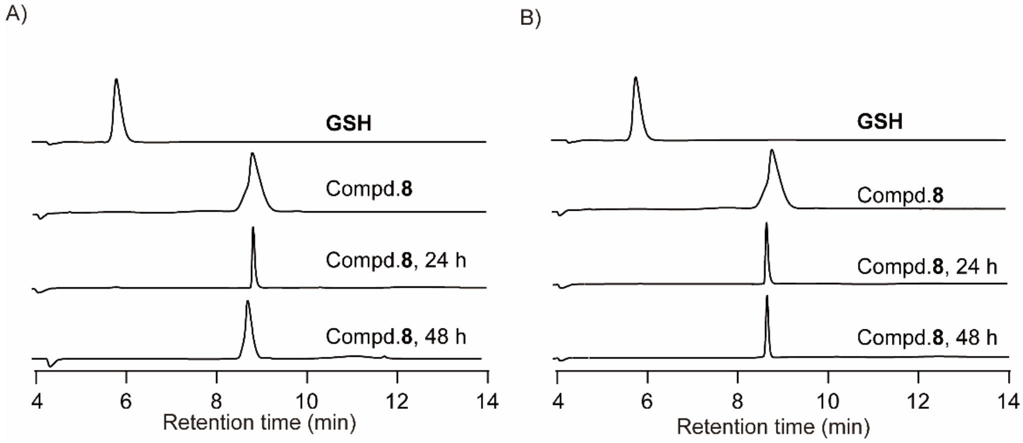

Figure S24 Stability of compound **8** in phosphate buffer analyzed by HPLC. (A) Compound **8** in 0.1 M phosphate buffer (pH 7.3) was monitored by HPLC at 24 and 48 h time points. (B) Compound **8** in 0.1 M carbonate buffer (pH 9.5) was monitored by HPLC at 24 and 48 h time points.

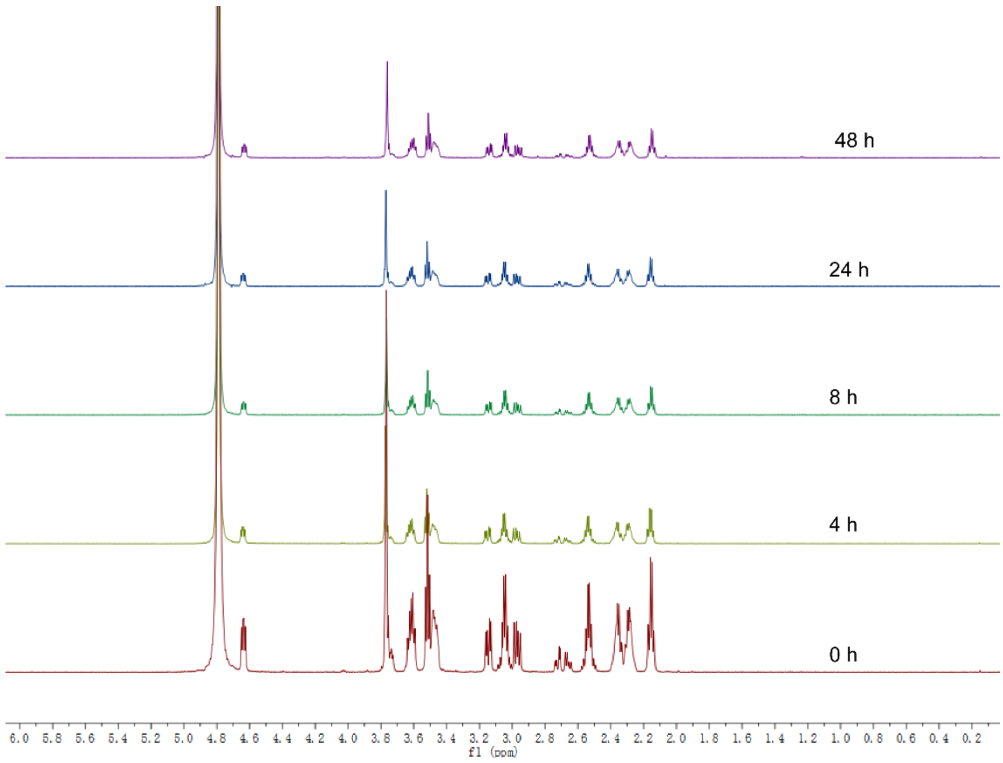

Figure S25 ^{1}H-NMR of compound **8** in phosphate buffer (pH 7.3) at different time points (0/4/8/24/48 h).

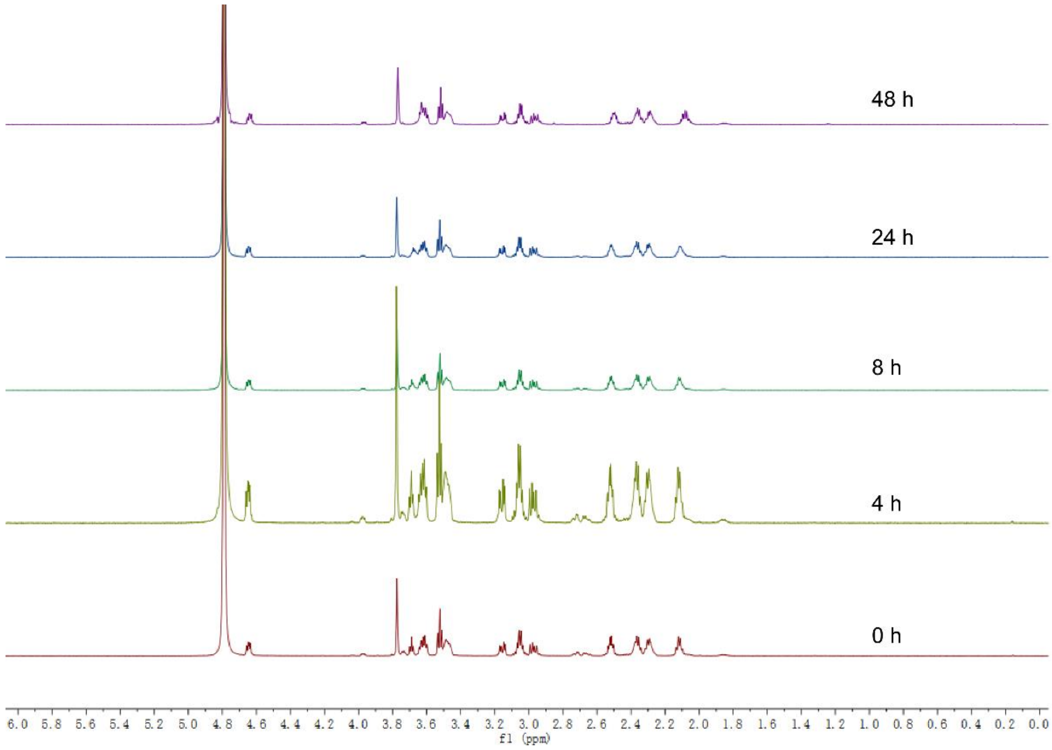

Figure S26 ^{1}H-NMR of compound **8** in carbonate buffer (pH 9.5) at different time points (0/4/8/24/48 h).

12.2 Stability study of sulfonium nucleosomes

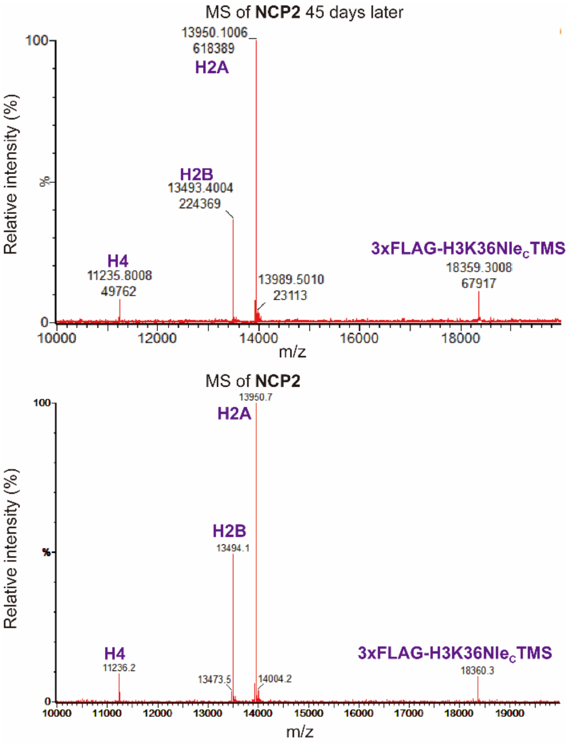

Figure S27 NCP2 in storage buffer was monitored by HR-ESI after 45 days.

13 Expression and purification of LEDGF and NSD2

Glutathione *S*-transferase GST-LEDGF (residues 1-100) was cloned into a pGEX-4T-1 vector and GST-NSD2 (residues 208-368) was constructed in pGEX-6P-1 vector. Protein expression and purification were performed as previously described.[4] Briefly, recombinant proteins were overexpressed in *Escherichia coli* strain BL21(DE3) induced by 0.2 mM isopropyl-β-D-1-thiogalactopyranoside (IPTG) at 16 °C for 16 h. Cells were collected and resuspended in lysis buffer (20 mM Tris, 150 mM NaCl, 1 mM DTT, 0.2 mM PMSF, pH 7.5) and sonicated. After cell lysis and centrifugation, proteins were loaded onto equilibrated glutathione-Sepharose 4B resin. After several washings, proteins were eluted with 10 mM glutathione buffer. Protein purity was analyzed by SDS-PAGE and the target proteins were dialyzed against storage buffer (20 mM Tris, 150 mM NaCl, 1 mM DTT, pH 7.5). Proteins were concentrated and quantified and finally stored at -80 °C.

14 Binding affinity between readers and sulfonium nucleosomes

All MicroScale Thermophoresis (MST) experiments were performed on a Monolith MT.115 machine with medium MST power and 20% excitation power in Nano-RED mode. The ligand proteins were serially diluted in PCR tubes with MST buffer (50 mM HEPES, 50 mM KCl, 2 mM BME, 0.1% Tween 20, pH 7.5). Then equal volume of fluorophore-labeled nucleosome was added and mixed with a final concentration of 20 nM. The mixture was incubated at room temperature for 10 min and the samples were transferred into capillaries (Monolith NT.115, NanoTemper, Germany). Capillaries were arranged in order of concentration and their fluorescence signal changes were detected and analyzed by MO. Affinity software to calculate the dissociation constants (K_d).

15 Crosslinking to H3K36me3 readers with sulfonium nucleosomes

Nucleosomes (final concentration: 0.5 μM) were incubated with H3K36me3 reader proteins (final concentration: 2 μM) in crosslinking buffer (100 mM HEPES, 150 mM NaCl, pH 7.5) on ice for 10 min. Then the samples were transferred to a 96-well plate for 302 nm (305 nm filter) irradiation for 20 min with energy of 7200×100 $\mu J/cm^2$ on ice. The crosslinking samples were analyzed by western blot with FLAG-HRP antibody (Yeasen, 30502ES60) and GST-HRP antibody (Abclonal, AE027).

16 HR-MS and NMR data

16.1 MS data of sulfoniums (ESI-TOF)

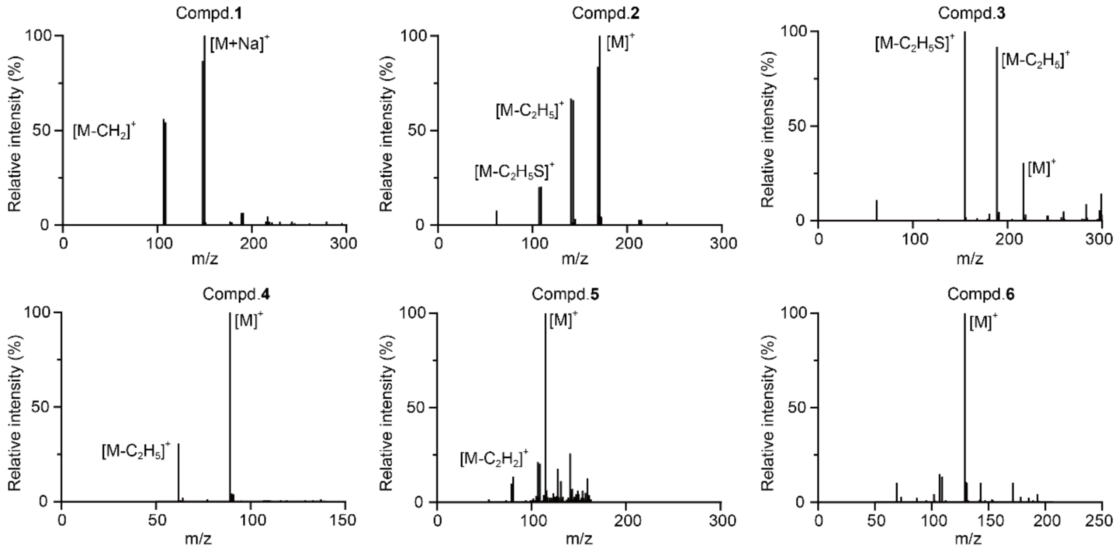

16.2 MS data of peptides (MALDI-TOF and ESI-TOF)

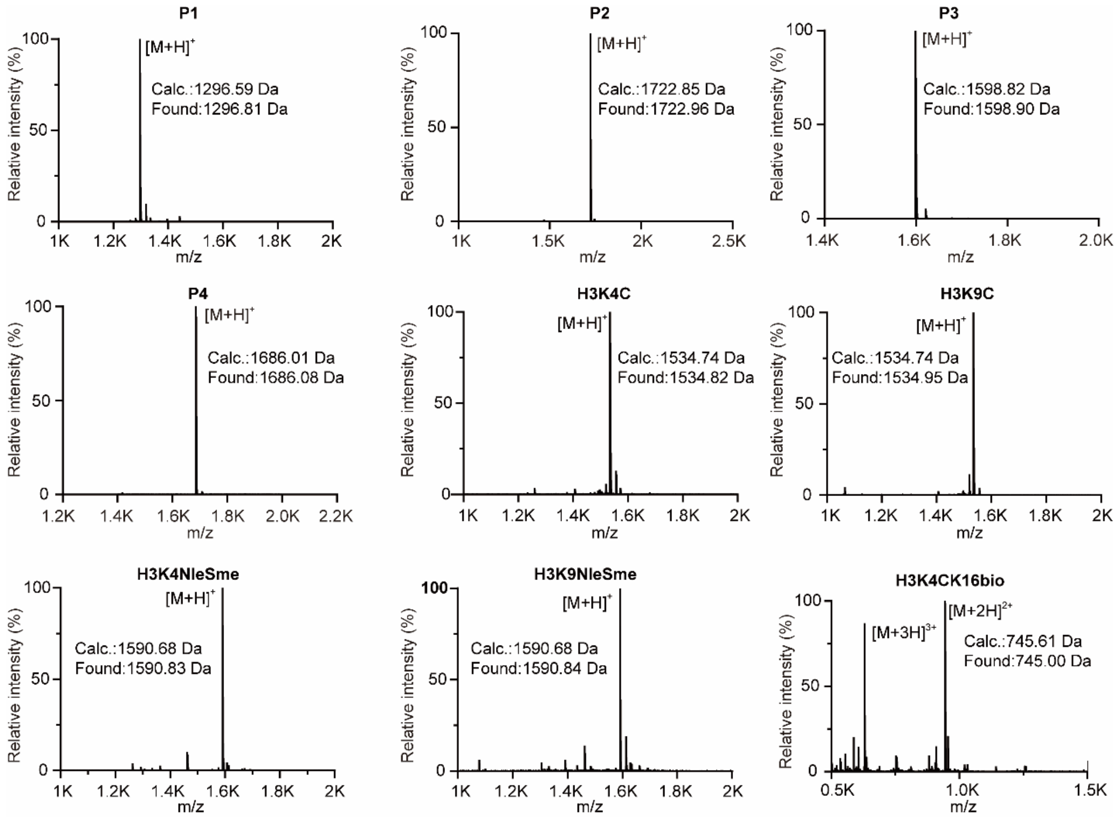

16.3 NMR of sulfoniums (^1H NMR and ^{13}C NMR)

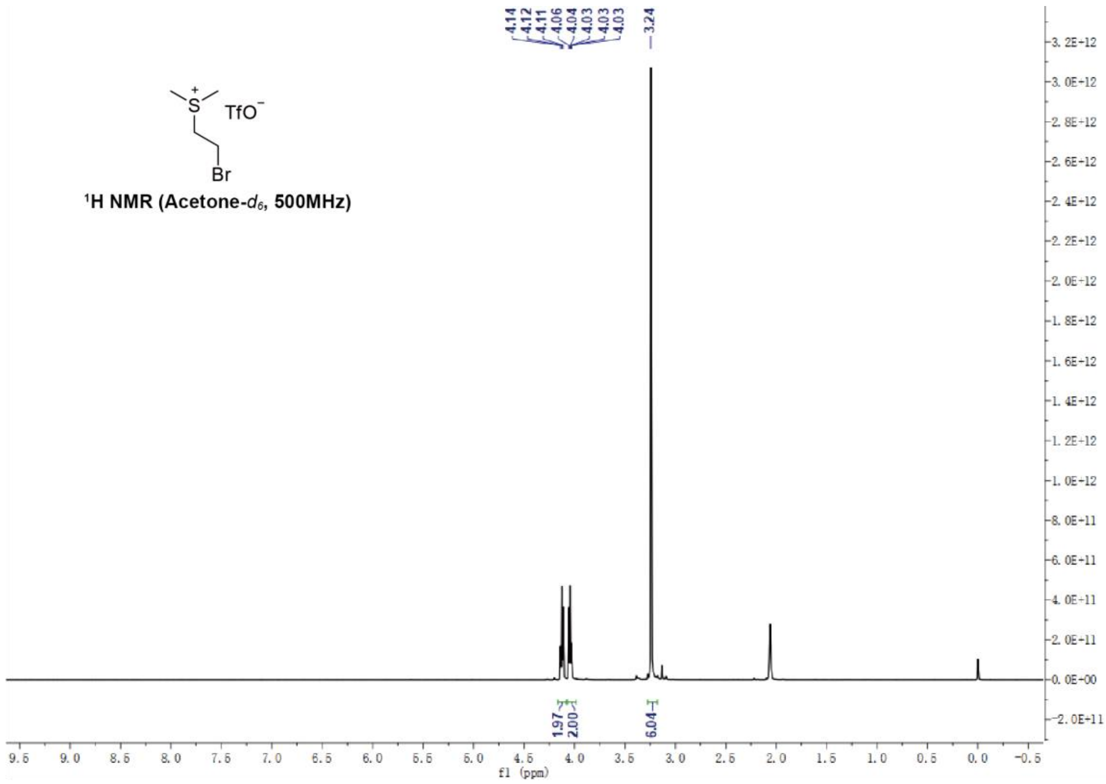

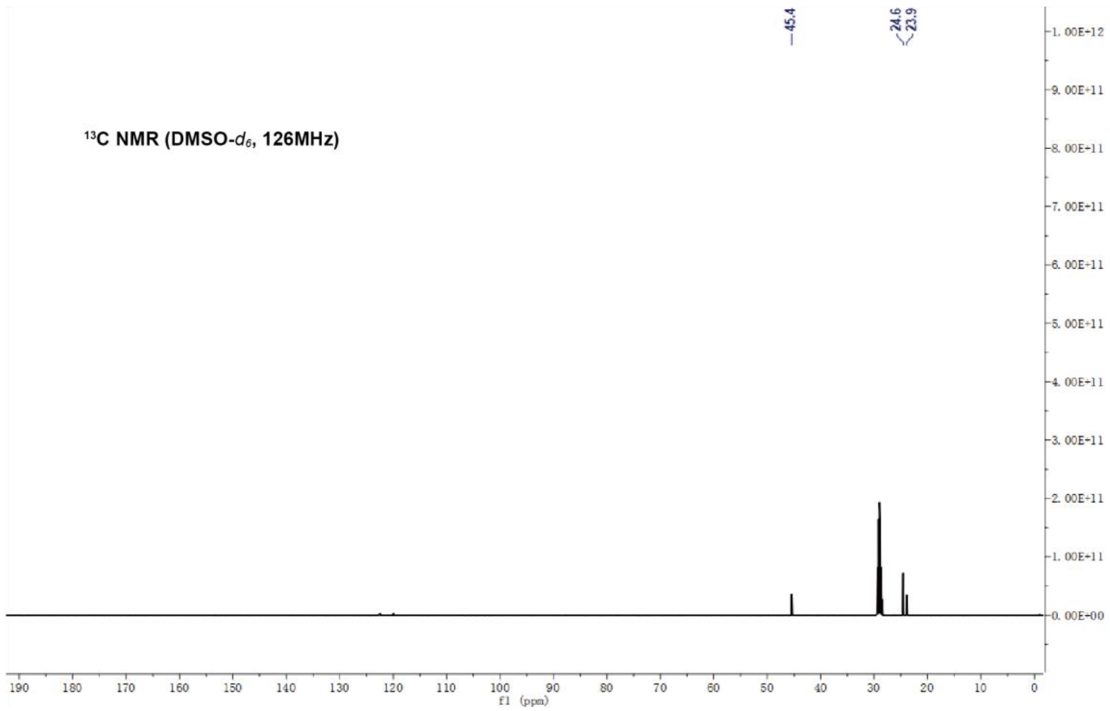

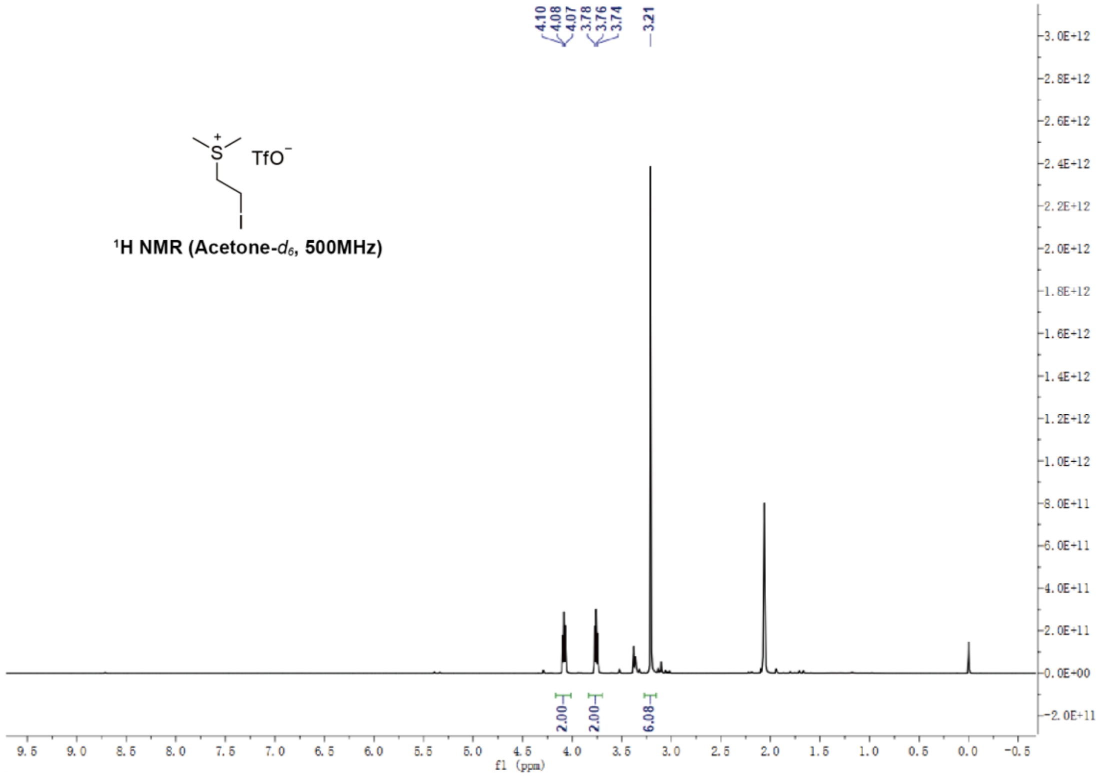

TfO$^-$
^{1}H NMR (Acetone-d_6, 500MHz)
4.10
4.08
4.07
3.78
3.76
3.74
3.21
2.00
2.00
6.08
f1 (ppm)

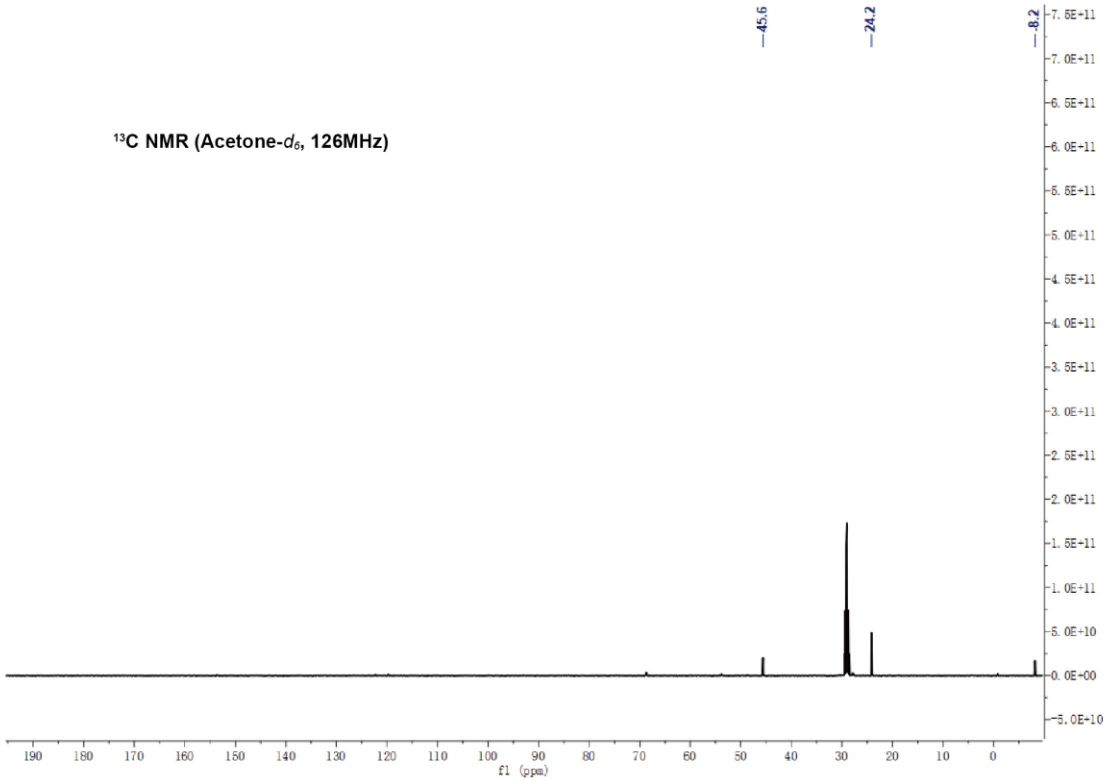

^{13}C NMR (Acetone-d_6, 126MHz)
45.6
24.2
-8.2
f1 (ppm)

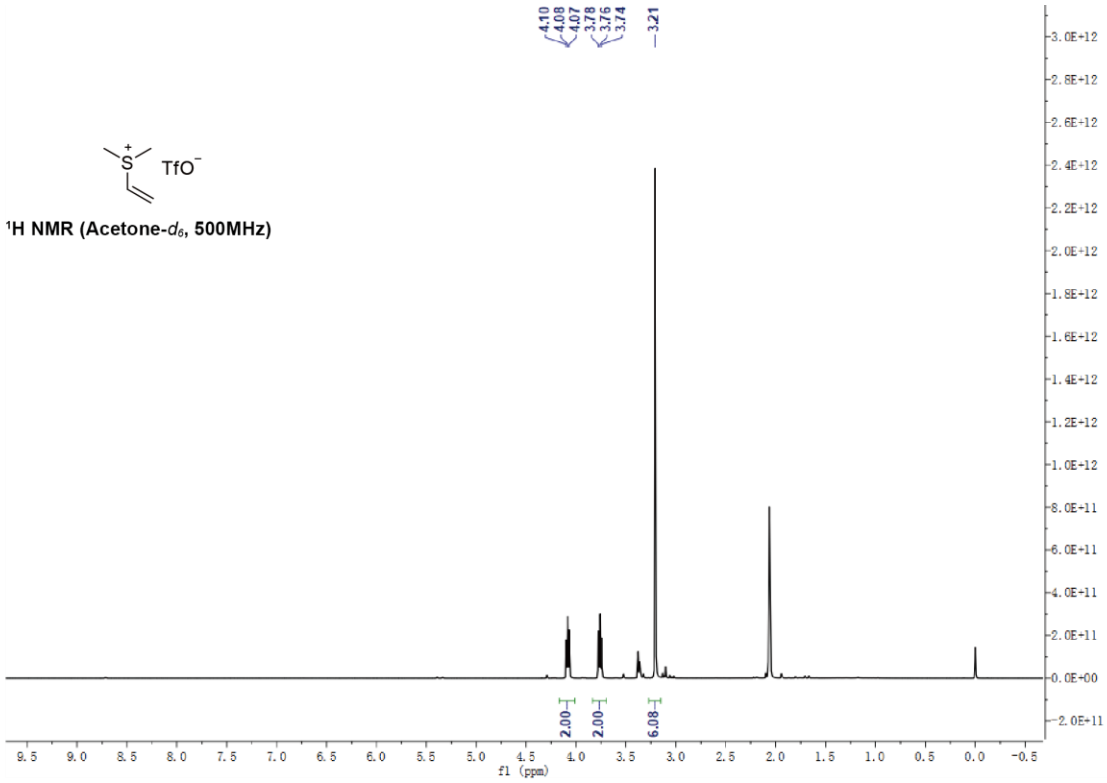

^{1}H NMR (Acetone-d_6, 500MHz)

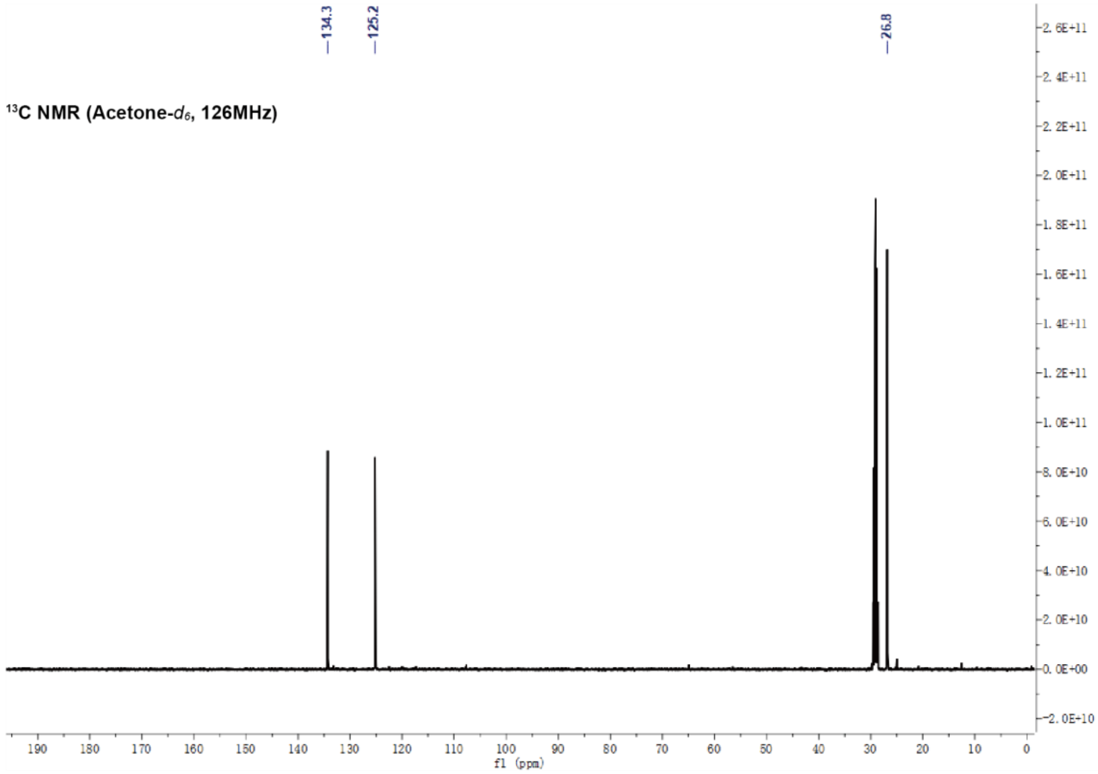

^{13}C NMR (Acetone-d_6, 126MHz)

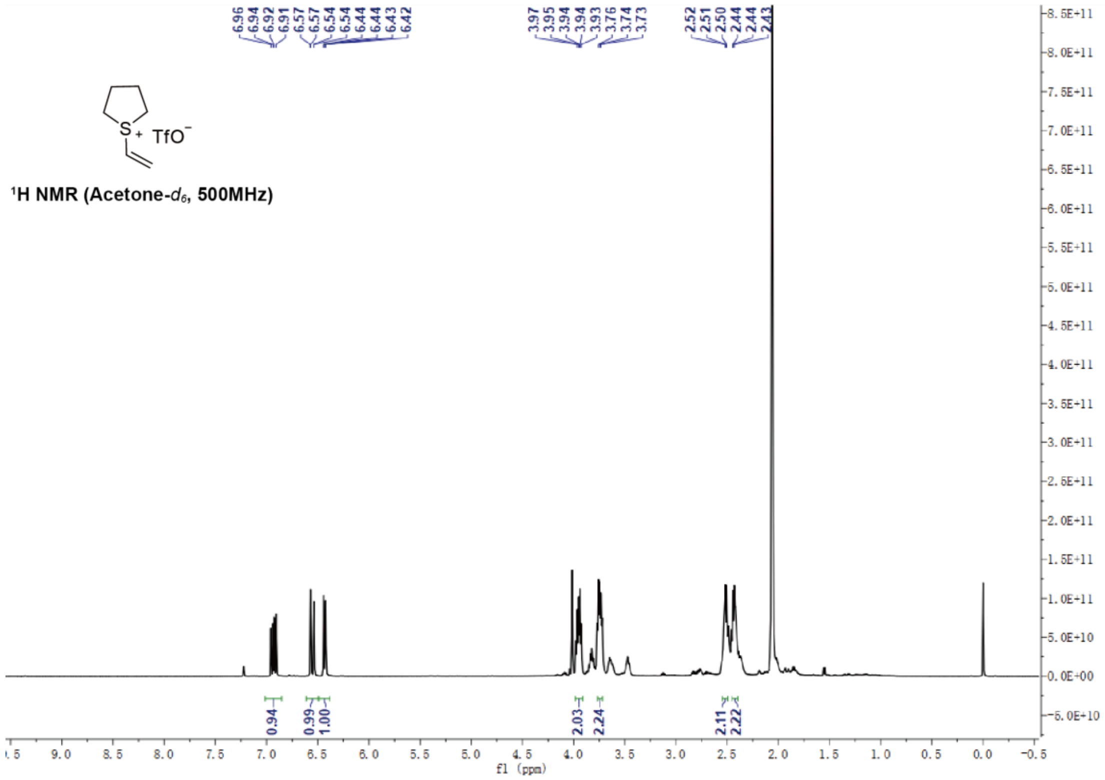
TfO⁻
^{1}H NMR (Acetone-d_6, 500MHz)
6.96 6.94 6.92 6.91 6.57 6.57 6.54 6.54 6.44 6.44 6.43 6.42
3.97 3.95 3.94 3.94 3.93 3.76 3.74 3.73
2.52 2.51 2.50 2.44 2.44 2.43
0.94 0.99 1.00 2.03 2.24 2.11 2.22
f1 (ppm)

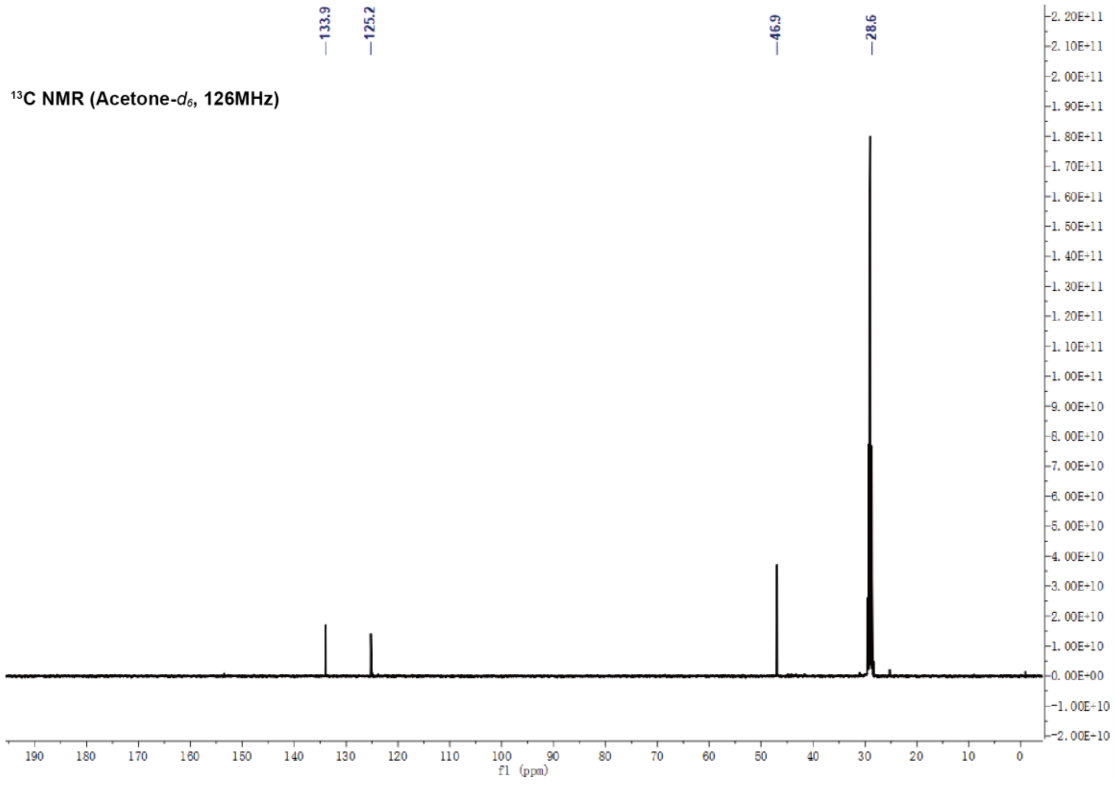
^{13}C NMR (Acetone-d_6, 126MHz)
133.9
125.2
46.9
28.6
f1 (ppm)

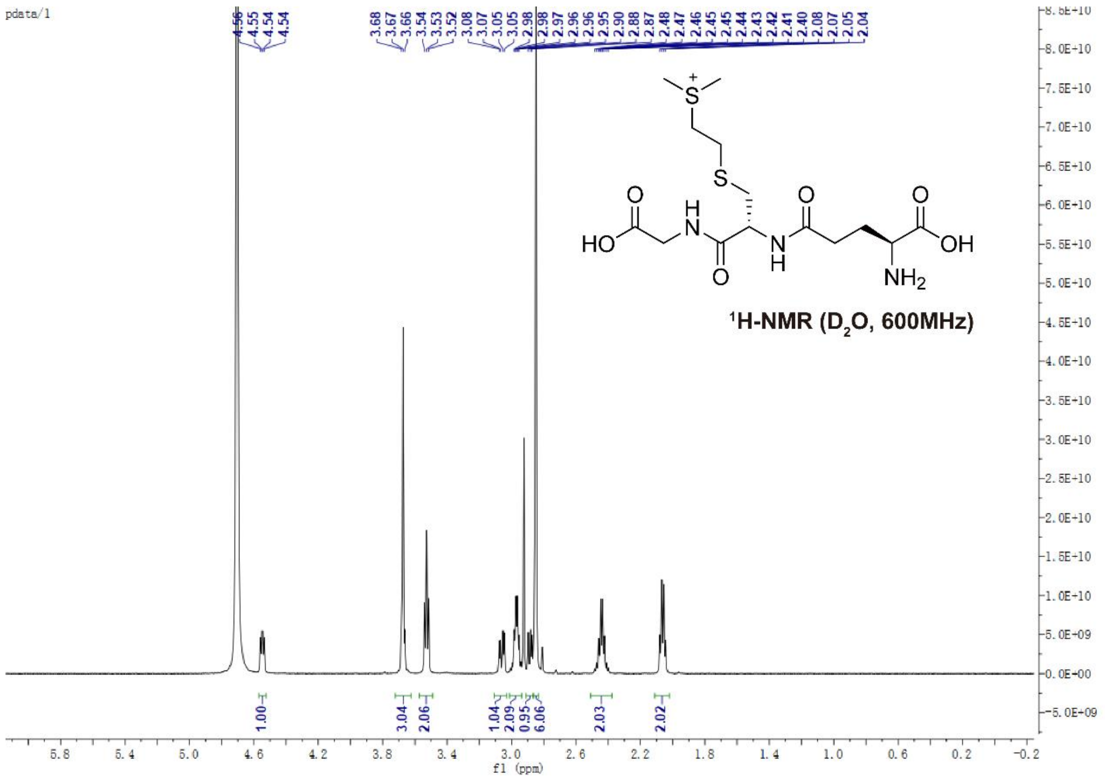
pdata/1
HO
OH
NH2
1H-NMR (D2O, 600MHz)
f1 (ppm)

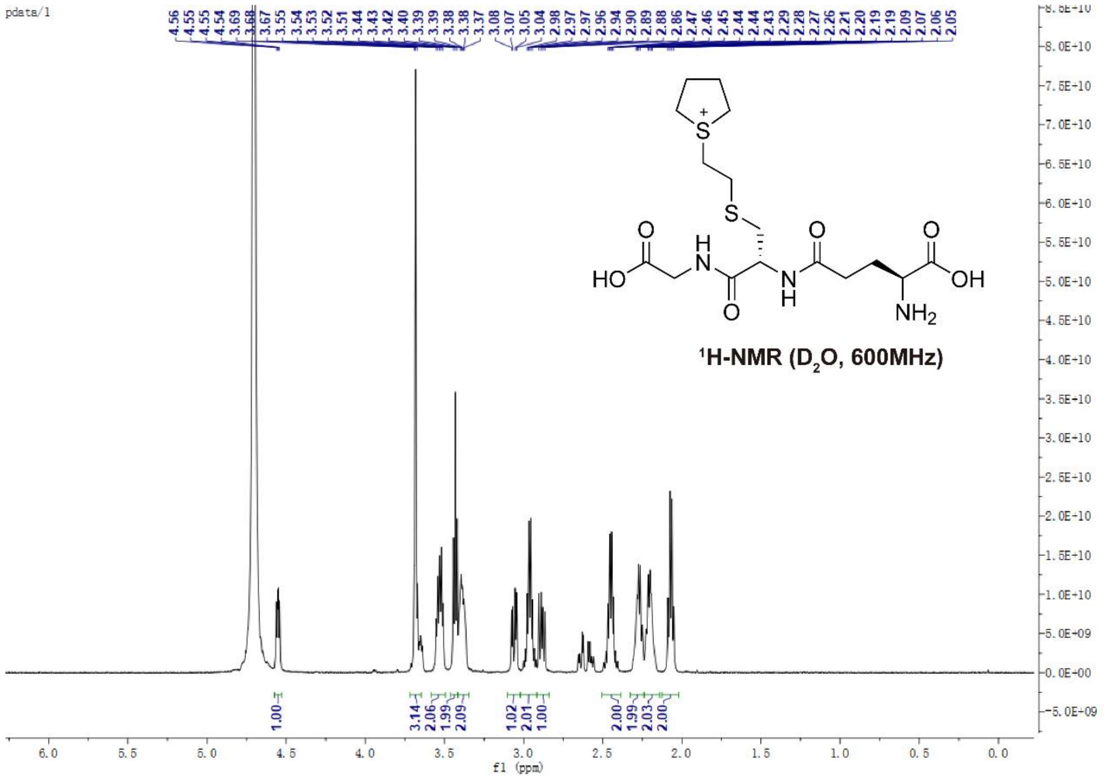
pdata/1
HO
OH
NH2
1H-NMR (D2O, 600MHz)
f1 (ppm)

17 References

1. F. Feng, Y. Gao, Q. Zhao, T. Luo, Q. Yang, N. Zhao, Y. Xiao, Y. Han, J. Pan, S. Feng, L. Zhang and M. Wu, *Nat. Chem.*, 2024, **16**, 1267-1277.
2. Q. Yang, Y. Gao, X. Liu, Y. Xiao and M. Wu, *Angew. Chem. Int. Ed.*, 2022, **61**, e202209945.
3. M. D. Simon, F. Chu, L. R. Racki, C. C. de la Cruz, A. L. Burlingame, B. Panning, G. J. Narlikar and K. M. Shokat, *Cell*, 2007, **128**, 1003-1012.
4. Y. Xiao, K. Zou, J. Yang and M. Wu, *Cell Rep. Phys. Sci.*, 2023, **4**, 101638.
